# Evolution and Introductions of Influenza A Virus H1N1 in a Farrow-to-Finish Farm in Guatemala

**DOI:** 10.1128/spectrum.02878-22

**Published:** 2022-12-07

**Authors:** Lucia Ortiz, Ginger Geiger, Lucas Ferreri, David Moran, Danilo Alvarez, Ana Silvia Gonzalez-Reiche, Dione Mendez, Daniela Rajao, Celia Cordon-Rosales, Daniel R. Perez

**Affiliations:** a Poultry Diagnostic and Research Center, Department of Population Health, College of Veterinary Medicine, University of Georgia, Athens, Georgia, USA; b Department of Microbiology and Immunology, Emory University School of Medicine, Atlanta, Georgia, USA; c Centro de Estudios en Salud, Universidad del Valle de Guatemala, Guatemala City, Guatemala; d Department of Genetics and Genomic Sciences, Icahn School of Medicine at Mount Sinai, New York, New York, USA; Changchun Veterinary Research Institute

**Keywords:** commercial farm, surveillance, Guatemala, influenza, molecular epidemiology, swine influenza

## Abstract

Commercial swine farms provide unique systems for interspecies transmission of influenza A viruses (FLUAVs) at the animal-human interface. Bidirectional transmission of FLUAVs between pigs and humans plays a significant role in the generation of novel strains that become established in the new host population. Active FLUAV surveillance was conducted for 2 years on a commercial pig farm in Southern Guatemala with no history of FLUAV vaccination. Nasal swabs (*n* = 2,094) from fattening pigs (6 to 24 weeks old) with respiratory signs were collected weekly from May 2016 to February 2018. Swabs were screened for FLUAV by real-time reverse transcriptase PCR (RRT-PCR), and full virus genomes of FLUAV-positive swabs were sequenced by next-generation sequencing (NGS). FLUAV prevalence was 12.0% (95% confidence interval [CI], 10.6% to 13.4%) with two distinct periods of high infection. All samples were identified as FLUAVs of the H1N1 subtype within the H1 swine clade 1A.3.3.2 and whose ancestors are the human origin 2009 H1N1 influenza pandemic virus (H1N1 pdm09). Compared to the prototypic reference segment sequence, 10 amino acid signatures were observed on relevant antigenic sites on the hemagglutinin. The Guatemalan swine-origin FLUAVs show independent evolution from other H1N1 pdm09 FLUAVs circulating in Central America. The zoonotic risk of these viruses remains unknown but strongly calls for continued FLUAV surveillance in pigs in Guatemala.

**IMPORTANCE** Despite increased surveillance efforts, the epidemiology of FLUAVs circulating in swine in Latin America remains understudied. For instance, the 2009 H1N1 influenza pandemic strain (H1N1 pdm09) emerged in Mexico, but its circulation remained undetected in pigs. In Central America, Guatemala is the country with the largest swine industry. We found a unique group of H1N1 pdm09 sequences that suggests independent evolution from similar viruses circulating in Central America. These viruses may represent the establishment of a novel genetic lineage with the potential to reassort with other cocirculating viruses and whose zoonotic risk remains to be determined.

## INTRODUCTION

Influenza A viruses (FLUAV) infect a wide range of avian and mammalian hosts, including humans. The virus genome is composed of 8 segments of negative single-stranded RNA corresponding to 6 internal (PB2, PB1, PA, NP, M, and NS) and 2 surface (hemagglutinin [HA] and neuraminidase [NA]) gene segments. Zoonotic FLUAV infections are relatively common, and it is accepted that influenza pandemics have resulted from zoonotic FLUAV strains (1). FLUAV infections in swine have a significant economic impact on swine production due to losses caused by the disease. Swine farms present high animal density and provide an environment for close contact between animals and humans. Such environments facilitate the initial spillover between species and transmission (2). Interspecies transmission events of FLUAVs between humans and pigs play a significant role in the generation of novel reassortant strains that transmit among humans and/or swine populations (3). Recent studies suggest that the introduction of human-origin FLUAVs into pigs is a major driver in the independent evolution of FLUAV lineages depending on geographic location (4). The emergence of the 2009 H1N1 influenza pandemic virus (H1N1 pdm09) in Latin America with potential undetected circulation in pigs for several years (5) highlights the need to improve influenza surveillance in these understudied regions to timely detect strains of zoonotic and/or pandemic concern.

In Central America, Guatemala is the country with the largest swine industry. Most of the swine production in Guatemala occurs in farrow-to-finish systems. According to the last census in 2021 by the Ministry of Agriculture, Livestock, and Food of Guatemala, the swine population was estimated to be 294,479 pigs, of which 32,518 were breeding stock (6). Circulation of FLUAV of human origin has been documented previously in swine populations in Guatemala (7); however, only limited genetic data is available. In this study, we conducted active surveillance to better understand the molecular epidemiology of FLUAV in swine populations in commercial pig farms in Guatemala (in the absence of vaccination). Phylogenetic analyses of all gene segments were performed to identify the potential origin of sequenced viruses. *In silico* characterization of the HA and NA was carried out to identify amino acid variants and molecular markers associated with viral resistance.

## RESULTS

### Association between FLUAV infection and male weaning pigs with fever.

**FIG 1:**
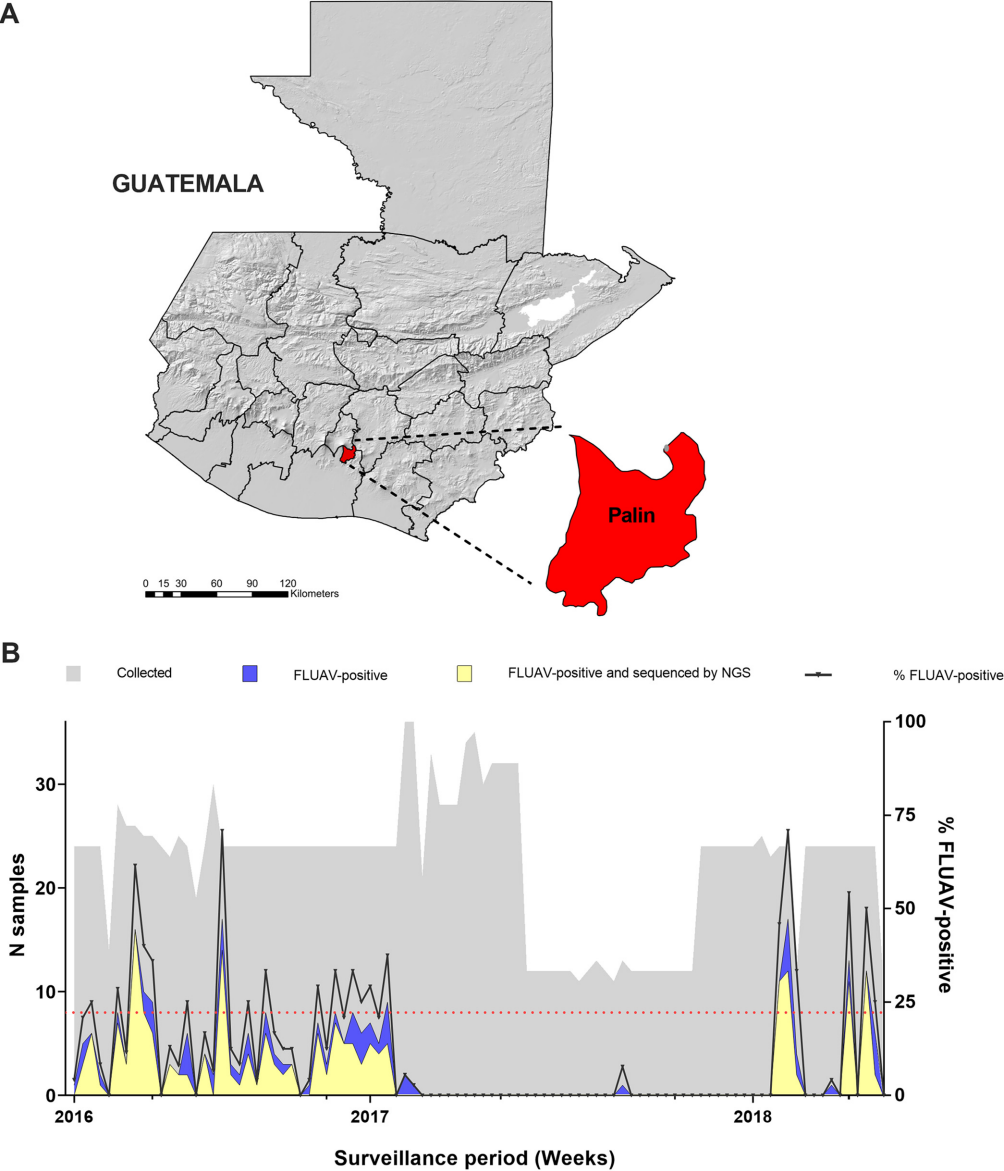
Location of study site (A) and number of collected FLUAV-positive and sequenced samples per week (B) during a 2-year FLUAV surveillance in a commercial farm in Guatemala. Sampled site represents the largest swine-producing region in the country with a history of FLUAV circulation. Red dotted line indicates the minimum number of samples required per week to achieve the desired sample size. Two high infection periods were detected by RRT-PCR, one from May 2016 to February 2017 and another one from January 2018 to April 2018.

From May 2016 to April 2018, we performed active FLUAV surveillance in a commercial farrow-to-finish farm located in Palin, Escuintla, ~60 miles away from the Southern Pacific coast of Guatemala (Fig. 1A).

The survey included 31,093 pigs associated to 3,113 cases of respiratory disease. Nasal swabs tested positive for FLUAV by real-time reverse transcriptase PCR (RRT-PCR) in 251 out of 2,094 samples, with an estimated prevalence of 12.0% (95% confidence interval [CI], 10.6% to 13.4%). Two periods of high FLUAV infection were detected, one from May 2016 to February 2017 and another from January 2018 to April 2018 with a prevalence of 18.7% (95% CI, 16.4% to 21.2%) and 17.8% (95% CI, 14.2% to 22.1%), respectively (Fig. 1B). Between these two periods, only one FLUAV-positive sample was detected in September 2017. The mean age of the pigs in the farm was 10.5 ± 6.6 weeks. Samples were collected from weaning pigs (4 to 10 weeks old) to adult pigs (>20 weeks old) (Table 1). FLUAV was detected in pigs as young as 5 weeks and as old as 21 weeks. The mean age of FLUAV-positive pigs was 8.0 ± 2.4 weeks. Factors such as age (weaning pigs odds ratio [OR] 95% = 4.1 [1.3, 20.7]), sex (male OR95% = 1.4 [1.0, 1.8]), and rectal temperature [fever OR95% = 2.7 (2.0, 3.6)] were associated with FLUAV positivity. Borderline association was found for animal density, 201 to 300 animals per pen (OR95% = 2.0 [1.0, 5.0]) and FLUAV detection. All animals presented coughing at the time of sampling.

**TABLE 1:** Risk factors associated with FLUAV detection by RRT-PCR in sampled pigs in Guatemala during 2016 to 2018

Characteristic	Total collected samples (%) (*n* = 2,094)	FLUAV positive (%) (*n* = 251)	FLUAV negative (%) (*n* = 1,843)	OR^*b*^ (95% IC)	*P* value
Sex					
Female	997 (47.6)	103 (41.0)	894 (48.5)	Referent	
Male	1,097 (52.4)	148 (59.0)	949 (51.5)	1.4 (1.0, 1.8)	0.0262
Age					
Weaning (4–10 wks)	1,468 (70.1)	232 (92.4)	1,236 (67.1)	4.1 (1.3, 20.7)	0.0098
Juvenile (11–17 wks)	307 (14.7)	11 (4.4)	296 (16.1)	0.8 (0.2, 4.7)	0.7618
Semi-adult (18–20 wks)	250 (11.9)	5 (2.0)	245 (13.3)	0.4 (0.1, 3.0)	0.2695
Adult (>20 wks)	69 (3.3)	3 (1.2)	66 (3.5)	Referent	
Rectal temp^*a*^					
<39.7	1,689 (80.7)	162 (64.5)	1,527 (82.9)	Referent	
>39.8	405 (19.3)	89 (35.5)	316 (17.1)	2.7 (2.0, 3.6)	0.0000
Density (animals/pen)					
<100	108 (5.2)	8 (3.2)	100 (5.4)	Referent	
101–200	339 (16.2)	24 (9.6)	315 (17.1)	1.0 (0.4, 2.5)	0.9084
201–300	782 (37.3)	110 (43.8)	672 (36.5)	2.0 (1.0, 5.0)	0.0558
301–400	810 (38.7)	107 (42.6)	703 (38.1)	1.9 (0.9, 4.7)	0.0871
>400	55 (2.6)	2 (0.8)	53 (2.9)	0.5 (0.0, 2.5)	0.3428

a Normal rectal temperature range for pigs: 38.7–39.8 (41).

b OR, odds ratio.

### Swine-origin FLUAVs in Guatemala show a separation from other contemporary human and swine H1N1 pdm09 detected in Central America.

From the 251 FLUAV-positive samples, 157 (62.5%) amplified at least one gene segment by multisegment RT-PCR (MS-RT-PCR). These samples were subsequently sequenced by next-generation sequencing (NGS), producing 57 complete genomes (53 from the 2016 to 2017 period and 4 from the 2018 period). The total number of full-length sequences by segment and the number of unique open reading frame (ORF) sequences are shown in Table 2. Best blast hits per all segments are shown in Table S1 in the supplemental material. To build the phylogenetic trees, only one representative from identical sequences was used, resulting in 273 unique nucleotide sequences (225 from the 2016 to 2017 period and 48 from the 2018 period; accession numbers can be found in Table S2 in the supplemental material).

**TABLE 2:** Total number of sequences obtained from sampled pigs in Guatemala during 2016 to 2018

Segment	No. of total full-length sequences	No. of ORF nucleotide sequences^*a*^		No. of unique amino acid sequences	
		2016–2017	2018	2016–2017	2018
PB2	106	86 (38)	20 (5)	14	4
PB1	71	68 (29)	4 (2)	8	2
PA	122	91(39)	31(5)	14 (PA), 3 (PA-X), 13 (PA-N155), 13 (PA-N182)	1 (PA), 1 (PA-X), 1 (PA-N155), 1 (PA-N182)
HA	144	106 (35)	38 (10)	12	6
NP	140	103 (25)	37 (8)	8	3
NA	137	101 (27)	36 (11)	11	6
M	157	116 (15)	41 (4)	6 (M1), 9 (M2)	1 (M1), 1 (M2)
NS	143	104 (17)	39 (3)	10 (NS1), 9 (NEP)	2 (NS1), 2 (NEP)
TOTAL	1,020	775 (225)	246 (48)		

a The number of unique ORF sequences used for phylogenetic analyses are indicated in parentheses.

Phylogenetic analyses of all gene segments show 2 clusters, one from samples collected from May 2016 to February 2017 and the other from samples collected in 2018. Within each cluster, all gene segments showed >99% sequence identity. Both clusters share an ancestor derived from the H1N1 pdm09 FLUAV lineage. The HA segments of all samples belonged to the FLUAV H1 swine clade 1A.3.3.2 derived from the H1N1 pdm09 FLUAV lineage using the “swine H1 clade classification tool” of the Influenza Research Database (IRD) (8).

**FIG 2:**
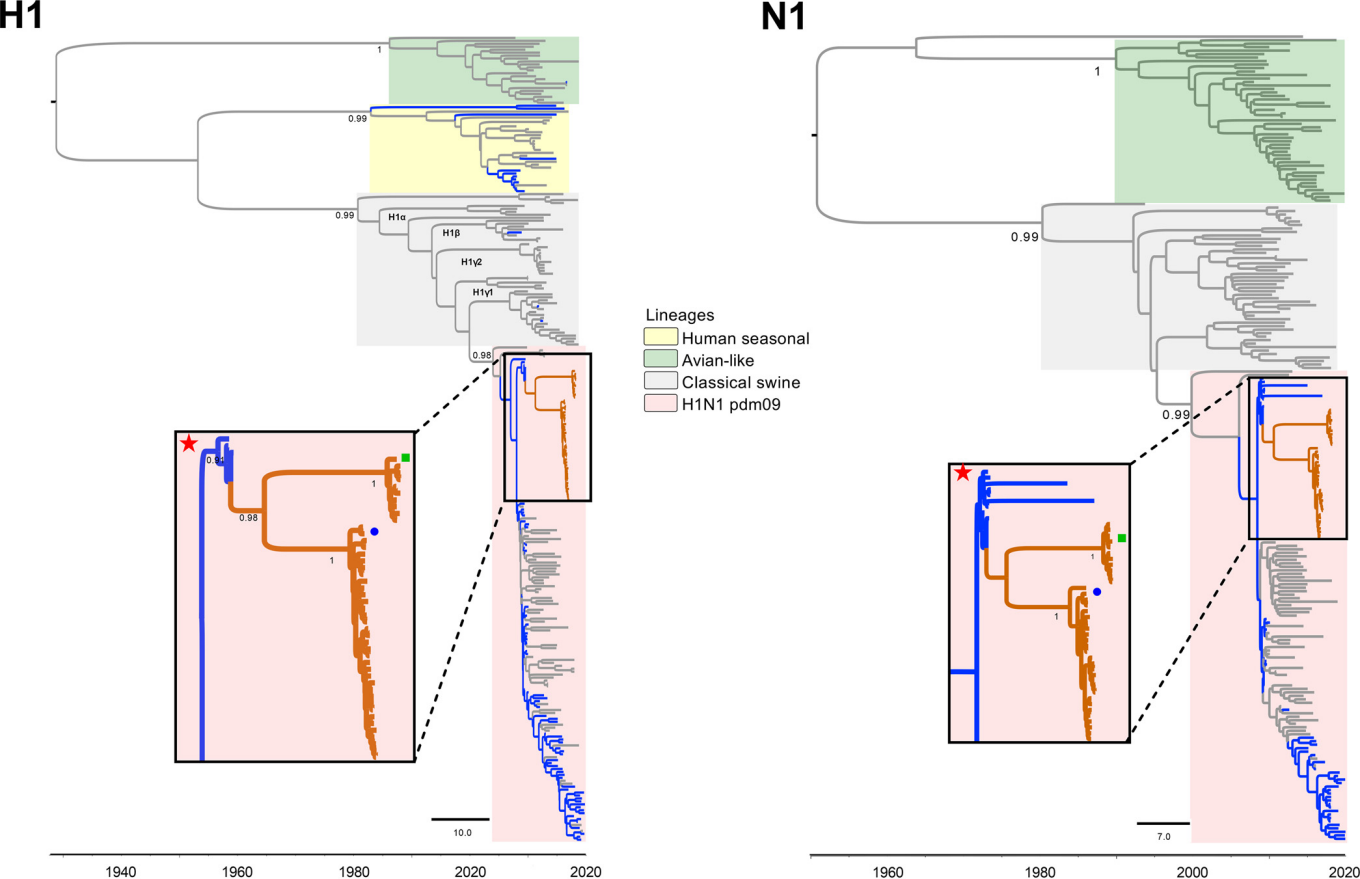
Surface gene (H1 and N1) time scaled MCC trees inferred for H1N1 pdm09 from swine and human coding sequences (2009 to 2019) for 273 (H1) and 229 (N1) sequences. Reference strain A/California/04/2009 is highlighted with a star symbol. Reference sequences were included to identify different lineages. Branches associated with viruses isolated from swine and human are shown in gray and blue, respectively. Branches of swine viruses from Guatemala are shown in orange. Identical sequences of swine viruses from Guatemalan were removed. The swine viruses from Guatemala are marked with a blue circle (May 2016 to February 2017 cluster) and a green rectangle (2018 cluster). Posterior probabilities >0.9 are included for key nodes.

**FIG 3:**
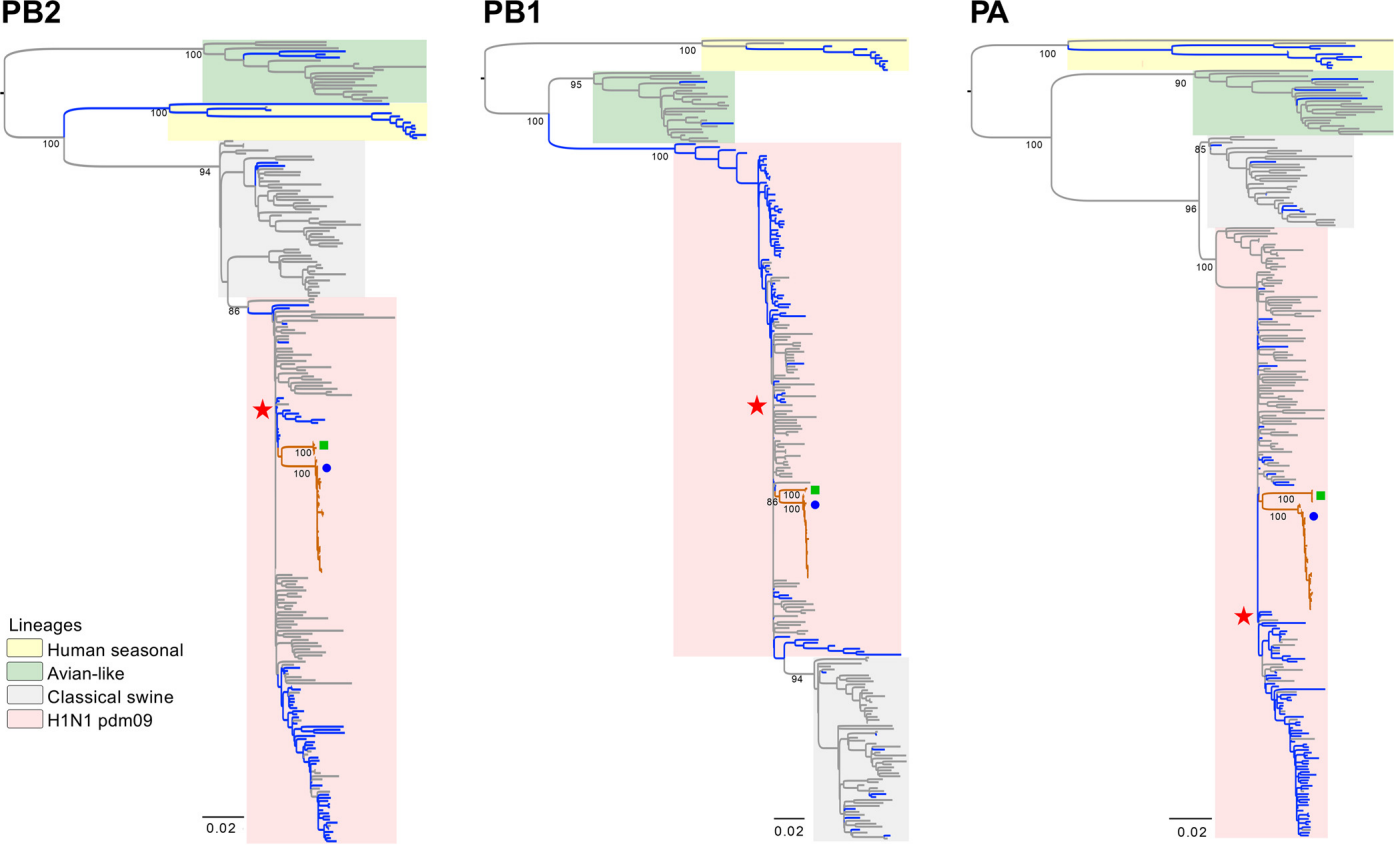
Internal gene (PB2, PB1, PA) phylogenetic inference for H1N1 pdm09 from swine and human coding sequences (2009 to 2019) for 259 (PB2), 270 (PB1), and 289 (PA) sequences. Maximum likelihood phylogenetic inference using the best-fit model. Reference sequences were included to identify different lineages. Reference strain A/California/04/2009 is highlighted with a star symbol. Branches associated with viruses isolated from swine and human are shown in gray and blue, respectively. Branches of Guatemalan viruses are shown in orange. Identical sequences of Guatemalan viruses were removed. The swine viruses from Guatemala are marked with a blue circle (May 2016 to February 2017 cluster) and a green rectangle (2018 cluster). Bootstrap values of >70% are included for key nodes.

**FIG 4:**
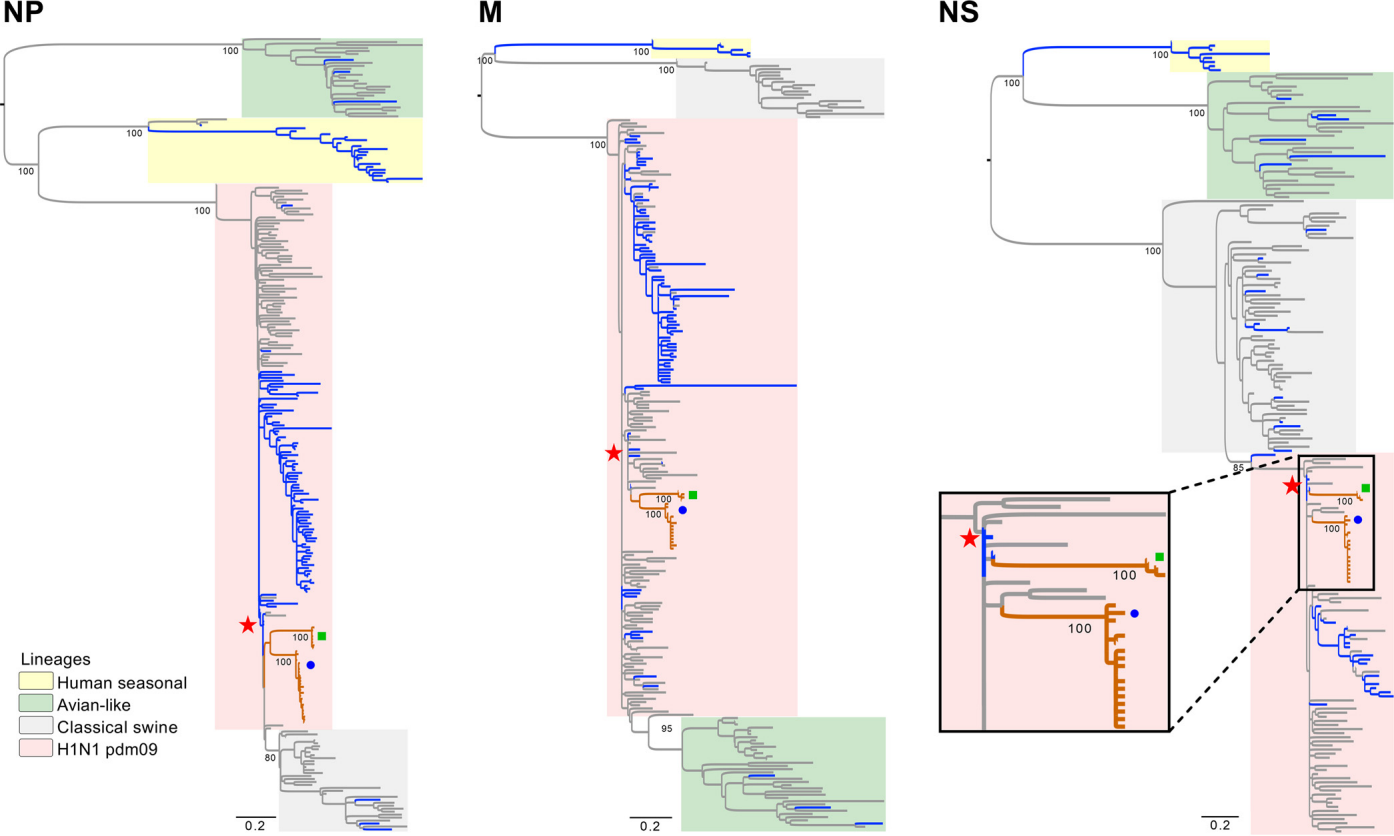
Internal gene (NP, M1, and NS1) phylogenetic inference for H1N1 pdm09 from swine and human coding sequences (2009 to 2019) for 267 (NP), 247 (M1), and 195 (NS1) sequences. Maximum likelihood phylogenetic inference using the best-fit model. Reference strain A/California/04/2009 is highlighted with a star symbol. Branches associated with viruses isolated from swine and human are shown in gray and blue, respectively. Branches of Guatemalan viruses are shown in orange. Identical sequences of Guatemalan viruses were removed. The swine viruses from Guatemala are marked with a blue circle (May 2016 to February 2017 cluster) and a green rectangle (2018 cluster). Bootstrap values of >70% are included for key nodes.

The HA and NA phylogenies of the Guatemalan FLUAVs show clear separation from other contemporary human H1N1 pdm09 FLUAVs and all swine FLUAVs detected in Central America whose sequences are available in the IRD and GISAID databases (Fig. 2). The HA and NA gene segments of Central American human origin FLUAV isolates from 2009 seem to be the most closely related ancestors of the HA and NA segments of the swine FLUAVs from Central America even though the samples in this study were collected during 2016 through 2018. It is estimated that these viruses were introduced early during the A(H1N1)pdm09 pandemic in ~2011.4 2009.6 to 2013.4 95% high posterior density [HPD] for phylogenetic analyses of the internal genes (PB2, PB1, PA, NP, and M) showed similar clustering within H1N1 pdm09 FLUAV in 2009 (Fig. 3 and 4). Interestingly, the NS gene segment was shown in two clusters, consistent with possibly two independent introductions (Fig. 4).

### Five amino acid signatures were fixed on relevant antigenic sites on the HA compared to the Ca04 reference sequence.

**FIG 5:**
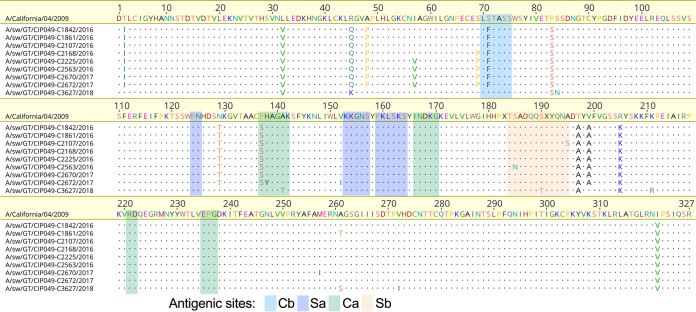
Distribution of amino acid mutations in the HA1 domain (H1 numbering without the signal peptide) of unique H1N1 pdm09 swine viruses from Guatemala aligned to A/California/04/2009 (GenBank accession no. GQ117044.1). Dots represent amino acids identical to the reference strain. The antigenic sites (Cb, Sa2, Ca1, and Sb) are shown in colored boxes.

Amino acid differences in HA observed in swine H1N1 pdm09 FLUAVs from Guatemala were compared to the reference strain A/California/04/2009 (H1N1) (Ca04) (Fig. 5; see also Fig. S1 in the supplemental material). The HA ORF encoded the same cleavage site sequence indistinguishable from the Ca04 strain (PSIQSR’GLF). In the rest of the HA ORF, up to 39 amino acid (aa) differences were observed compared to the Ca04 reference sequence (Fig. S1), 25 of those on the HA1 globular head with 10 falling within antigenic sites Cb (S71F), Ca2 (P137S, H138Y, A141T), Ca1 (G170E, R205K, E235G), and Sb (S185N, S190T, A195S) (H1 numbering, mature protein). Minor variant frequency analysis revealed other mutations on the HA1 globular head in different animals (A141E, L161P, T184A, T184I, D187G, Q188R, and E235K), with allele frequency ranging from 2.3% to 36.3% in at least 8 viruses from the 2016 to 2017 period.

**FIG 6:**
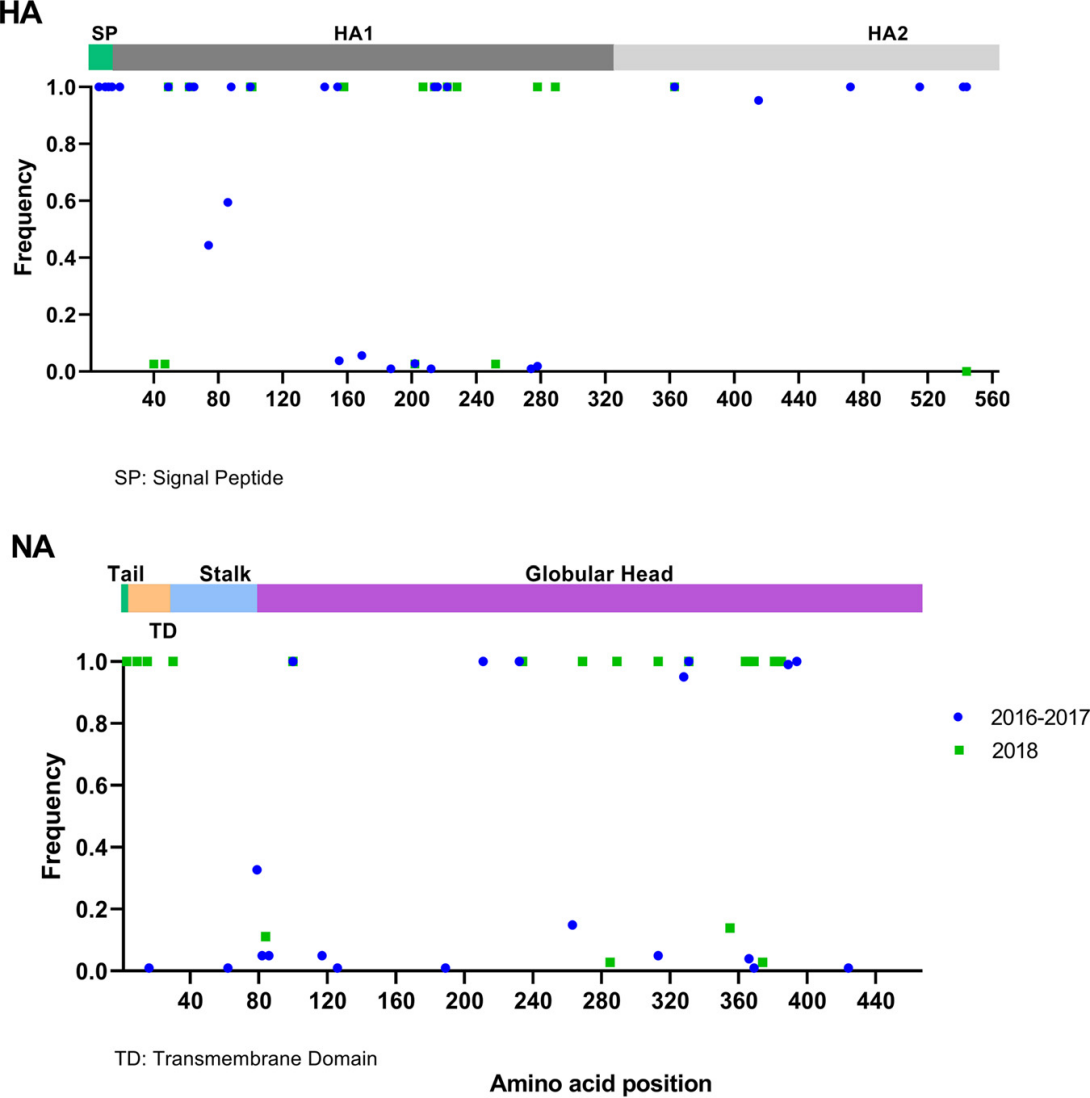
Distribution and frequency of amino acid point mutations in proteins of surface genes (HA and NA) from H1N1 pdm09 swine viruses from Guatemala compared to those of the reference strain A/California/04/2009 (GenBank accession no. GQ117044.1 and MN371610.1). Known protein domains or important sites of pdm09 haplotypes available in public databases are represented by colored blocks. Amino acid position is shown in the *x* axis.

Most amino acid differences in the HA1 region occurred at high frequency (>0.95) (Fig. 6). Five of these amino acid positions were in common among the HA ORFs from the two high FLUAV infection periods (P83S, T197A, R205K in antigenic site Ca1, I321V, and D346E). The HA R45Q and the HA R45K were fixed in viruses from the 2016 to 2017 and 2018 periods, respectively (Fig. S1). The HA L32V was the most common signature in viruses from both infection periods (>100 HA sequences), except for one virus each from 2016 and 2018 (L32M and L32I, respectively). S71F (Cb) and P137S (Ca2) were fixed in viruses from the 2016 to 2017 and A141T and S190T in the 2018 period.

### Amino acid signatures elsewhere in predicted ORFs of swine FLUAVs from Guatemala.

In the NA ORF, 36 amino acid differences were found compared to the N1 NA from the Ca04 reference strain (see Fig. S1), but most were found fixed in NA sequences from the 2018 period (18 amino acid signatures in 35 sequences). In contrast, only 5 amino acid signatures were identified in >90% out of 101 sequences in the NA sequences from the 2016 to 2017 period. Only the Y100H signature was in common among sequences between the two periods, whereas the NA K331R and NA K331N were fixed in strains from the 2016 to 2017 and 2018 periods, respectively. None of these differences were present in either in the catalytic site (118, 151, 152, 224, 276, 292, 371, and 406; N2 numbering) or the framework residues (119, 156, 178, 179, 198, 222, 227, 274, 277, 294, and 425; N2 numbering) (9), and no amino acid changes in known drug binding sites were found (Fig. 6).

**FIG 7:**
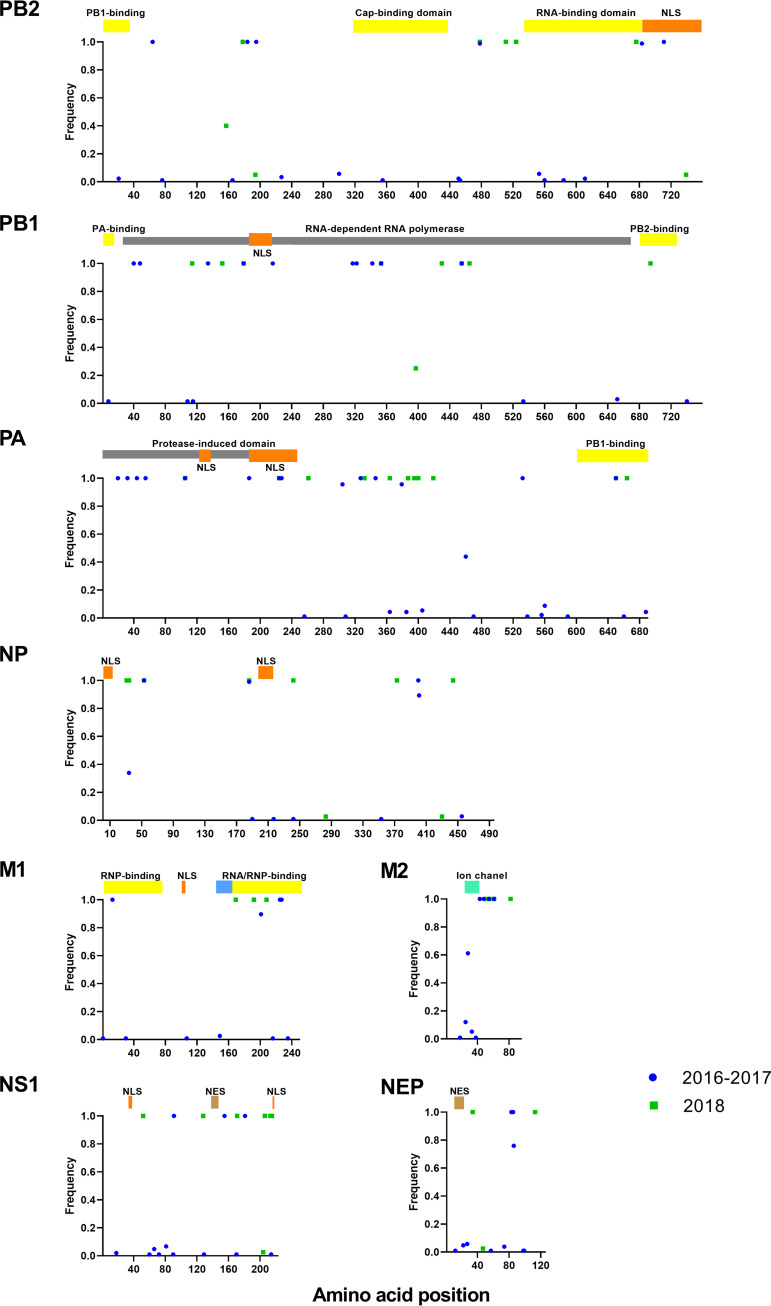
Distribution and frequency of amino acid point mutations in PB2, PB1, PA, NP, M1, M2, NS1, and NEP from swine H1N1 pdm09 FLUAVs from Guatemala compared to those of the reference strain A/California/04/2009 (GenBank accession no. MN371615.1, MN371613.1, MN371611.1, MN371617.1, FJ969513.1, and FJ969514.1). Known protein domains or important sites of pdm09 haplotypes available in public databases are represented by colored blocks.

In the internal gene segments, eight substitutions that appeared in both periods were found in PB1, PA, NP, and M2 (Fig. 7; see also Fig. S2 and S3 in the supplemental material). Compared to the Ca04 reference, the PB1 fixed mutations I179M, K353R, and N455D are located on predicted RNA-dependent RNA polymerase motifs. PA presented fixed mutations in the NLS region (P224S) and the PB1 binding domain (Y650F), while NP showed a mutation overlapping the PB2 binding and RNA binding domains (D53E). M2 showed two fixed mutations found in both periods, located at amino acid positions 55 and 61 (F55I and R61K). All swine origin FLUAV Guatemalan viruses presented a truncated form of the PB1-F2 protein of 11 aa, due to premature stop codons at positions 12 and 58, in common with the Ca04 reference strain (10). All swine origin FLUAV Guatemalan viruses encode the PA-X functional protein (232 aa), as well as the ORFs for PA-N155 (562 aa) and PA-N182 (535 aa). No alternative fixed mutations were found in PA-X in either period compared to Ca04. In contrast, PA-N155 and PA-N182 ORFs show two mutations, P70S/Y498F and P43S/Y469F, respectively (see Fig. S4 in the supplemental material).

No mammalian-associated virulence markers in PB2 (E627K, D701N), PB1-F2 (N66S), NS (S42P, D92E, and V149A), and M2 (V27A) were found for any of the analyzed sequences. All swine origin FLUAV Guatemalan viruses showed the PA S409N signature predictive of increased virulence and the M2 S31N marker of amantadine resistance like other Eurasian swine lineage M segments (11–13).

## DISCUSSION

Close contact between humans and pigs in swine production systems may result in bidirectional transmission of different FLUAVs between the two species. Systematic surveillance of FLUAV in swine production farms provides a unique opportunity to study how these viruses may jump and adapt at the human-animal interface and identify novel strains that may become established in the new host population. Based on an extensive FLUAV weekly surveillance in pigs from 2016 to 2018, we detected an overall FLUAV prevalence of 12% in animals with respiratory disease, similar to previous studies in pigs in Guatemala (7). It must be noted that sick animals were identified and sampled based on observation of coughing (regardless of other signs), which may have resulted in underrepresentation of sampling and consequent underestimation of the FLUAV prevalence found in this study. In this study, weaning pigs (4 to 10 weeks old) showed the highest positivity rate of FLUAV and were associated with higher rates of infection, consistent with previous surveillance studies (7, 14). Factors such as sex (males) and fever were also associated with FLUAV positivity.

During the span of 2 years, we detected two high infection periods but not a seasonality pattern. Only the H1N1 subtype from the H1N1 pdm09 lineage was identified, despite other FLUAV subtypes being reported in humans and swine in Guatemala and Central America at the time of the surveillance period (7, 15). National records from Guatemala indicate minimal to zero circulation of H1N1 pdm09 FLUAVs in humans at the time of pig sampling for this study. This observation suggests a prior introduction of H1N1 pdm09 FLUAVs into pigs that remained endemic in the swine population in Guatemala. Serological studies should be incorporated to identify additional subtypes that might be circulating in the farm. Interestingly, a sharp reduction in FLUAV-positive samples was observed for approximately 12 months during most of 2017. This drop in detection could be explained by an increase in prevention and biosafety practices following the first year of the study (annual seasonal influenza vaccination of workers, quarantine of sick pigs, use of hand disinfectant and mask respirators by workers, and enforcement of the shower-in/shower-out procedures) as reported by farm owners. However, the reason for the subsequent increase in detections seen in 2018 remains unknown, although it is consistent with the notion of introduction of a virus strain through replacement animals brought into the farm. During this study, the farm owners reported that at least 30% of their replacement gilts came from neighboring farms. Another potential viral source might be piglets less than 4 weeks of age, which have been identified as shedders, while they were still with the sows (16). Unfortunately, at the time of the study, we were not able to sample pigs younger than 4 weeks of age. The role of these animals at this age deserves more in-depth studies.

Virus isolation was not performed during this study, instead FLUAV NGS was performed directly from original swabs. Using this approach, we obtained sequences from >75% of the FLUAV-positive samples and nearly 25% of complete genomes by NGS directly from the original swabs. This methodology allowed us to improve the number of characterized samples and reduce potential selection bias introduced by virus isolation prior to sequencing (17). Interestingly, all samples presented a high number of defective RNAs as was observed in agarose gels and later by the valley-like shape in the graphs of the polymerase genes (PB2, PB1, and PA) of the NGS coverage read maps (see Fig. S5 in the supplemental material). These particles are truncated forms of FLUAV generated by most viruses during virus replication that retain the terminal sequences necessary for virus packaging (18) and are mostly present in the polymerase segments (19, 20). The function is not fully understood, but it is hypothesized that they could play a role in maintaining low levels of replication of infectious virus (21).

Phylogenetic analyses showed that the swine origin FLUAV Guatemalan viruses are clearly segregated from other H1N1 pdm09 viruses in the Americas, suggesting independent evolution of these viruses after introduction and subsequent circulation in pigs, consistent with reports in other regions (3). At least two independent introductions were observed as noted with the separated clusters of Guatemalan samples. Interestingly, the viruses seemed to have been introduced in 2011 during the H1N1 pdm09 pandemic and persisted as separate clades for ~5 to 7 years before detection by our surveillance. Long persistence sustained prevalence of human-origin FLUAVs in environments with significant interaction between two FLUAV hosts might lead to the generation of strains of pandemic concern. Unfortunately, at the time of the study, it was not possible to sample farm workers. Futures studies need to be focused on the swine–human interface to compare phylogenetic relationships between viruses collected from these two hosts to determine the potential origin of these viruses and define their characteristics of evolution.

Little human seasonal influenza sequence data is available from Guatemala and Central America and much less swine FLUAV sequence data exists. This gap in part explains the results of the phylogenetic relationships and the lack of a clear explanation of the most closely related ancestors. Independent virus evolution events have been documented previously in pigs, particularly after introduction of human FLUAVs, showing long phylogenetic branches between the swine strains and their putative human FLUAV ancestor, as shown previously from other Latin American countries (4, 22). Here, we describe the circulation and evolution of H1N1 pdm09 lineage viruses in Guatemala that may represent the establishment of a novel genetic lineage with the potential to reassort with cocirculating viruses. The mutations found in relevant HA1 antigenic sites may lead to differences in antigenic relationships with other H1N1 pdm09 viruses of human or swine origin; however, the effect of these mutations and zoonotic risk remain to be determined. These observations highlight the need for increased and sustained influenza surveillance within understudied regions.

## MATERIALS AND METHODS

### Collection site.

We selected one commercial farrow-to-finish farm located in Palin, Escuintla (Fig. 1A) with 13,044 pigs, and of these, 1,233 were breeding stock at the time of the study. The farm production system is divided into boars, sows, replacements, piglets, and growing areas, located on different premises within the farm. Artificial insemination, gestation, and farrowing are performed routinely in the farm. The site did not routinely vaccinate against influenza at the time of the study. Boars, sows, and replacements are in the same facility shared with quarantine, insemination, gestation, replacements, and maternity areas. Piglets are weaned at 4 weeks of age. After weaning, pigs are moved to a different area where they spend 6 weeks (from week 4 to week 10). Afterward, pigs are moved to the finish stage, where they spend approximately 12 more weeks (from week 10 to week 22) to get ready for market. The production cycle of each pig takes approximately 22 weeks. For the past 10 years, the farm reported a history of FLUAV exposure in swine.

### Sample collection.

A total of 2,094 nasal swabs from fattening pigs (6 to 24 weeks old) and sows with respiratory signs were collected from May 2016 to April 2018. Pigs were identified and sampled by farm workers when presenting clinical signs of respiratory disease, including coughing, sneezing, and nasal and/or ocular discharges, to ensure that sampled animals were those most likely to be affected by and shedding the virus (23). A minimum of 32 nasal swabs were collected monthly to detect at least 1 positive sample based on a prevalence of 10% or higher, with a 95% confidence, and in a group size of ≥600 (24) according to the FAO guidelines (23). The farm was sampled twice per week, ensuring that the minimum sample size calculated per month was met. Pigs were tattooed with a unique identifier to avoid resampling. Rectal temperature, sex, respiratory signs, and pen density of each animal were recorded at the time of sampling. Nasal swabs were collected and preserved in 3 mL of virus transport medium (VTM) with antibiotics and antimycotics as described previously (7). Sampling of animals was conducted under approved animal use protocols from the Ministry of Agriculture, Livestock, and Food of Guatemala (MAGA), and the protocols were reviewed and approved by the Institutional Animal Use and Care Committee of the University del Valle de Guatemala.

### RNA extraction.

Virus RNA from nasal swabs was extracted from 50 μL of supernatant using the MagMAX‐96 AI/ND virus RNA isolation kit (Ambion, Austin, TX) according to the manufacturer's instructions. All extracted RNA was stored at −70°C until further use.

### FLUAV detection by RRT-PCR.

Swabs were screened in duplicate for FLUAV by real-time reverse transcriptase PCR (RRT-PCR) targeting the matrix gene as described previously (7, 25, 26). Briefly, QuantiTect Probe RT-PCR (reverse transcription PCR) kit (Qiagen, Hilden, Germany) was used to perform RRT-PCRs in the ABI 7300 real-time PCR system (Applied Biosystems, Foster City, CA). Each reaction contained 12.5 μL of kit-supplied 2× RT-PCR master mix, 10 pmol of each primer, 0.3 μM probe, 0.25 μL of kit-supplied enzyme mix, 6.5 U RNase inhibitor, and 8 μL of RNA template. Thermal cycling conditions were as follows: one cycle of reverse transcription at 50°C for 30 min and 94°C for 15 min, followed by 45 cycles of denaturation at 94°C for 1 s and combined annealing and extension at 60°C for 27 s.

### Multisegment amplification.

Multisegment amplification of FLUAV genes (MS-RTPCR) was performed from RNA extracted from all FLUAV-positive swabs as described previously (27) with minor modifications. Briefly, 2.5 μL of extracted RNA was used as a template in a 25-μL MS-RTPCR reaction (Superscript III high-fidelity RT-PCR kit; Thermo Fisher) using Opti1-F1 (0.06 μM), Opti1-F2 (0.14 μM), and Opti1-R1 (0.2 μM) primers (17). The cycling conditions were as follows: 55°C for 2 min, 42°C for 1 h, 5 cycles (94°C for 30 s, 44°C for 30 s, 68°C for 3 min), followed by 31 cycles (94°C for 30 s, 57°C for 30 s, 68°C for 3 min). Final extension was at 68°C for 10 min. The MS-RTPCR final product was analyzed in 1% agarose gel to corroborate whole-genome amplification.

### Sequencing and genome assembly.

MS-RTPCR products were sequenced using the Illumina platform as described previously (17) with minor modifications. Briefly, amplicons from MS-RTPCR reactions were cleaned by 0.45× Agencourt AMPure XP magnetic beads (Beckman Coulter) according to the manufacturer’s protocol and eluted in 30 μL of HyClone molecular biology water (Genesee Scientific). Amplicons were quantified using the Qubit buffer kit (Fisher Scientific) in Qubit 3.0 fluorometer (Thermo Fisher) and normalized to 0.2 ng/μL. Adaptors were added by tagmentation using the Nextera XT DNA library preparation kit (Illumina). The reaction was set as 60% of the suggested final volume. Samples were purified using 0.7× Agencourt AMPure XP magnetic beads and analyzed on a Bioanalyzer using a high sensitivity DNA kit (Agilent) to determine the distribution of fragment size. Libraries were pooled and normalized to 1 to 4 nM. After denaturation, the final loading concentration of the pooled libraries was 14 pM. Libraries were sequenced using the MiSeq reagent kit V2 300 cycles (Illumina). Genome assembly was performed using a customized pipeline developed at the Icahn School of Medicine at Mount Sinai (5).

### Minor variants detection.

Variant calling was performed as described previously (28) using LoFreq 2.1.3.1 (29) following the Genome Analysis Toolkit best practices (30).

### Phylogenetic analysis.

Independent phylogenetic analyses were performed for the surface genes (HA and NA) and internal genes (PB2, PB1, PA, NS, NP, and M) of the sequenced viruses. The additional FLUAV genome sequences from human- and swine-origin H1N1 pdm09-like viruses, as well as those of avian origin from the Americas and Eurasia (from 2009 to 2019) were downloaded from the Global Initiative on Sharing All Influenza Data (GISAID) (http://platform.gisaid.org) and the Influenza Research Database (IRD) (http://www.fludb.org). Sequences were aligned with MUSCLE 5.1 (31) and manually trimmed to keep the open reading frame (ORF) of the gene of interest. Background sequences were subsampled to remove identical sequences and those sequences with less than 80% of the total length of the ORF using SeqKit 0.16.1 bioinformatic tool (32). Representative sequences were randomly selected by region and host. Identical sequences obtained from this study were excluded, and only one representative of each sequence was used. The best-fit model of nucleotide substitution was determined for each gene using the Bayesian information criterion (BIC) obtained using jModelTest 2.1.10 (33). For the internal genes, phylogenetic trees were constructed using maximum likelihood (ML) inference methods, using RAxML 8.2.12 (34) under the general time reversible GTR+G nucleotide substitution model with 1,000 bootstrap replicates. Trees were run at least twice to confirm topology. Surface gene (H1 and N1) phylogenies were inferred by time-scaled phylogenetic analysis using the Bayesian Markov chain Monte Carlo (MCMC) approach as implemented in BEAST 1.10.4 (35, 36). A relaxed uncorrelated lognormal (UCLN) molecular clock was used, with a constant population size and a general-time reversible (GTR+G) model of nucleotide substitution. Three independent analyses of 50 to 100 million generations were performed to ensure convergence, sampling every 5,000 states. The burn-in percentage of each data set was identified using Tracer v1.7.1 (37), and after its removal, results were combined using LogCombiner v1.10.4 (35, 36). The MCC trees were annotated using TreeAnnotator v1.10.4 (35, 36). Outlier sequences were identified using TemEst v1.5.3 (38). When necessary, outlier sequences were removed, and the data sets were run again as described above. Nucleotide alignment, background sequences subsampling, BIC, and phylogenetic analyses (RaxML and Beast) were performed using the computational resources Sapelo2 available at The University of Georgia. The resulting trees were visualized in FigTree 1.4.4 and aesthetically modified using Inkscape v0.48.1 (https://inkscape.org). Additionally, standard nucleotide BLAST searches were used to find regions of local similarity between isolates obtained in this study and other sequences available in GenBank (39).

### Amino acid sequence analysis.

Nucleotide sequences were translated using SeqKit 0.16.1 bioinformatic tool (32) and visualized on Geneious Prime 2021.2.2 (https://www.geneious.com). The resulting amino acid sequences were compared to prototypic reference sequences to calculate the frequency of phenotypic markers for mammalian transmission/pathogenicity and identify specific motifs in the sequence.

### Statistical analysis.

The percentages of positive pigs detected by RRT-PCR were calculated by week, as the total number of positive detected by RRT-PCR by the total number of collected samples per week. To analyze the risk of FLUAV infection, odds ratios (ORs) were estimated using Stata 15.1 (40) and GraphPad Prism v.9.4.0 (https://www.graphpad.com).

### Data availability.

Nucleotide genomic sequences from all Guatemalan viruses have been deposited at the NCBI Database under accession numbers ON822118 to ON823137.
