# Supplementary material for: Evolution and Introductions of Influenza A Virus H1N1 in a Farrow-to-Finish Farm in Guatemala
Source: Microbiol Spectr. 2022 Dec 7;11(1):e02878-22. doi: 10.1128/spectrum.02878-22 (PMC9927084; doi:10.1128/spectrum.02878-22)
Supplement: Supplemental file 1 — Supplemental material. Download spectrum.02878-22-s0001.pdf, PDF file, 5.9 MB [file spectrum.02878-22-s0001.pdf]

Supplemental Material

Fig S1. HA (H1 numbering) and NA (N2 numbering) unique amino acids of H1N1 pdm09 swine viruses from Guatemala aligned to A/California/04/2009 (GenBank accession no. GQ117044.1 and MN371610.1). Dots represent amino acids identical to A/California/04/2009. The antigenic sites for HA (Cb, Sa2, Ca1 and Sb) are shown in green boxes; whereas viruses collected during May 2016 to February 2017 are shown in orange and those collected in 2018 in grey.

Fig S2. PB2, PB1, PA and NP unique amino acids of H1N1 pdm09 swine viruses from Guatemala aligned to A/California/04/2009 (GenBank accession no. MN371615.1, MN371613.1, MN371611.1 and MN371617.1). Dots represent amino acids identical to A/California/04/2009. Viruses collected during May 2016 to February 2017 are shown in orange and those collected in 2018 in grey.

Fig S3. M1, M2, NS1 and NEP unique amino acids of H1N1 pdm09 swine viruses from Guatemala aligned to A/California/04/2009 (GenBank accession no. FJ969513.1, FJ969514.1). Dots represent amino acids identical to A/California/04/2009. Viruses collected during May 2016 to February 2017 are shown in orange and those collected in 2018 in grey.

Fig S4. PA-X, PA-N155 and PA-N182 unique amino acids of H1N1 pdm09 swine viruses from Guatemala aligned to A/California/04/2009 (GenBank accession no. MN371611.1). Dots represent amino acids identical to A/California/04/2009. Viruses collected during May 2016 to February 2017 are shown in orange and those collected in 2018 in grey.

Fig S5. Coverage plot of sequenced samples. Gray lines show the coverage distribution from each individual sample. The red line depicts the geometric mean.

Table S1. Most closely related FLUAV gene for PB2, PB1, PA, HA NP, NA, M, and NS full-length genes of Guatemalan viruses obtained from sampled pigs during 2016-2018.

**Segment**	**2016-2017**			**2018**		
	**Identity (%)**	**Blast hit**	**GenBank accession no.**	**Identity (%)**	**Blast hit**	**GeneBank accession no.**
PB2	98.035	A/Ontario/156785/2009(H1N1)	CY076792.1	98.163	A/Ontario/156785/2009(H1N1)	CY076792.1
PB1	97.821	A/swine/Quebec/1698189/2014(H1N1)	KX571177.1	97.864	A/swine/Quebec/1698189/2014(H1N1)	KX571177.1
	97.821	A/Belgium/145-MA/2009(H1N1)	KJ867560.1	97.864	A/Belgium/145-MA/2009(H1N1)	KJ867560.1
	97.821	A/Singapore/ON1852/2009(H1N1)	CY123554.1	97.537	A/Santo Angelo/LACENRS-1537/2009(H1N1)	KY925240.1
PA	97.716	A/Igrejinha/LACENRS-1803/2009(H1N1)	KY926042.1			
	97.716	A/Teutonia/LACENRS-711/2009(H1N1)	KY926136.1			
	97.716	A/Santo Antonio da Patrulha/LACENRS-1649/2009(H1N1)	KY925446.1			
	97.716	A/Santa Cruz do Sul/LACENRS-2013/2009(H1N1)	KY925354.1			
	97.716	A/Porto Alegre/LACENRS-3434/2009(H1N1)	KY925771.1			
HA	97.133	A/Tenente Portela/LACENRS-2073/2009(H1N1)	KY925117.1	97.243	A/Porto Alegre/LACENRS-1786/2009(H1N1)	KY925200.1
	97.133	A/Sao Gabriel/LACENRS-1626/2009(H1N1)	KY925045.1			
	97.133	A/Singapore/ON129/2009(H1N1)	CY123459.1			
	97.133	A/Guangdong/55/2009(H1N1)	HQ011423.1			
	97.133	A/Singapore/GN285/2009(H1N1)	CY055303.1			
NP	98.211	A/California/04/2009(H1N1)	MK159424.1	97.7	A/California/04/2009(H1N1)	MK159424.1
	98.211	A/Giessen/6/2009(H1N1)	KC620387.1			
	98.211	A/Singapore/478/2009(H1N1)	CY069635.2			
NA	96.914	A/Mexico/IBT25/2009(H1N1)	MG856198.1	95.748	A/Mexico/IBT25/2009(H1N1)	MG856198.1
				95.748	A/Campina das Missoes/LACENRS-2882/2009(H1N1)	KY925041.1
				95.748	A/Singapore/ON1908/2009(H1N1)	CY123610.1
M	97.955	A/Singapore/GP1112/2010(H1N1)	JX309362.1	97.955	A/California/04/2009(H1N1)	MK159425.1
	97.955	A/swine/Taiwan/TD-4119B2/2009(H1N1)	GU324342.1	97.955	A/Hamburg/1094-MA/2009(H1N1)	KY472237.1
NS	98.09	A/California/04-223-MA/2009(H1N1)	KX136128.1	97.64	A/swine/Quebec/1683217/2014(H1N1)	KX571147.1
				97.64	A/Porto Alegre/LACENRS-2121/2009(H1N1)	KY926156.1
				97.64	A/Ontario/25389/2009(H1N1)	CY060546.1

HA

	# of identical sequences					2	23	30	32	45	48	57	69	Cb 71	83	84	129	Ca2 137	138	141	152	Ca1 170	Sb 185	190	195	197	199	Ca1 205	211	Ca1 235	257	261	272	321	346	398	455	498	525	527
**A/California/04/2009**		**V**	**Y**	**F**	**T**	**T**	**N**	**V**	**L**	**R**	**A**	**I**	**S**	**S**	**P**	**S**	**N**	**P**	**H**	**A**	**V**	**G**	**S**	**S**	**A**	**T**	**V**	**R**	**K**	**E**	**M**	**A**	**V**	**I**	**D**	**N**	**N**	**E**	**V**	**V**
A/Guatemala/1039/2009		.	.	.	.	.	.	.	I	K	.	.	.	.	S	.	.	.	.	.	.	.	.	.	.	A	.	K	.	.	.	.	.	V	.	.	.	.	.	.
A/sw/GT/CIP049-C1842/2016	5	A	N	L	I	I	.	.	V	Q	P	.	.	F	S	.	T	S	.	.	.	.	.	.	.	A	A	K	.	.	.	.	.	V	E	.	T	K	I	I
A/sw/GT/CIP049-C1861/2016	2	A	N	L	I	I	.	.	V	Q	P	.	.	F	S	.	T	S	.	.	.	.	.	.	.	A	A	K	.	.	.	T	.	V	E	S	T	K	I	I
A/sw/GT/CIP049-C1913/2016	35	A	N	L	I	I	.	.	V	Q	P	.	.	F	S	.	T	S	.	.	.	.	.	.	.	A	A	K	.	.	.	.	.	V	E	S	T	K	I	I
A/sw/GT/CIP049-C2107/2016	1	A	N	L	I	I	.	.	V	Q	P	.	.	F	S	.	T	S	.	.	.	.	.	.	S	A	A	K	.	.	.	.	.	V	E	S	T	K	I	I
A/sw/GT/CIP049-C2214/2016	16	A	N	L	I	I	.	.	V	Q	P	.	P	F	S	.	T	S	.	.	.	.	.	.	.	A	A	K	.	.	.	.	.	V	E	S	T	K	I	I
A/sw/GT/CIP049-C2356/2016	1	A	N	L	I	I	.	.	V	Q	P	V	P	F	S	.	T	S	.	.	.	E	.	.	.	A	A	K	.	.	.	.	.	V	E	S	T	K	I	I
A/sw/GT/CIP049-C2541/2016	3	A	N	L	I	I	.	.	V	Q	P	V	P	F	S	.	T	S	.	.	.	.	N	.	.	A	A	K	.	.	.	.	.	V	E	S	T	K	I	I
A/sw/GT/CIP049-C2547/2016	35	A	N	L	I	I	.	.	V	Q	P	V	P	F	S	.	T	S	.	.	.	.	.	.	.	A	A	K	.	.	.	.	.	V	E	S	T	K	I	I
A/sw/GT/CIP049-C2609/2016	1	A	N	L	I	I	.	.	M	Q	P	V	P	F	S	.	T	S	.	.	.	.	.	.	.	A	A	K	.	.	.	.	.	V	E	S	T	K	I	I
A/sw/GT/CIP049-C2632/2017	2	A	N	L	I	I	.	.	V	Q	P	V	P	F	S	.	T	S	.	.	I	.	.	.	.	A	A	K	.	.	.	.	.	V	E	S	T	K	I	I
A/sw/GT/CIP049-C2670/2017	1	A	N	L	I	I	.	.	V	Q	P	V	P	F	S	.	T	S	.	.	.	.	.	.	.	A	A	K	.	.	I	.	.	V	E	S	T	K	I	I
A/sw/GT/CIP049-C2671/2017	4	A	N	L	I	I	.	.	V	Q	P	V	P	F	S	.	T	S	Y	.	I	.	.	.	.	A	A	K	.	.	.	.	.	V	E	S	T	K	I	I
A/sw/GT/CIP049-C3618/2018	33	.	.	.	.	.	.	.	V	K	.	.	.	.	S	N	.	.	.	T	.	.	.	T	.	A	.	K	R	.	.	S	I	V	E	.	.	.	.	.
A/sw/GT/CIP049-C3646/2018	1	.	.	.	.	.	.	.	V	K	.	.	.	.	S	N	.	.	.	T	.	.	N	T	.	A	.	K	R	.	.	S	T	V	E	.	.	.	.	.
A/sw/GT/CIP049-C3649/2018	1	.	.	.	.	.	.	.	V	K	.	.	.	.	S	N	.	.	.	T	.	.	.	T	.	A	.	K	R	G	.	S	I	V	E	.	.	.	.	.
A/sw/GT/CIP049-C3661/2018	1	.	.	.	.	.	D	.	V	K	.	.	.	.	S	N	.	.	.	T	.	.	.	T	.	A	.	K	R	.	.	S	I	V	E	.	.	.	.	.
A/sw/GT/CIP049-C3841/2018	1	.	.	.	.	.	.	.	I	K	.	.	.	.	S	N	.	.	.	T	.	.	.	T	.	A	.	K	R	.	.	S	I	V	E	.	.	.	.	.
A/sw/GT/CIP049-C3853/2018	1	.	.	.	.	.	.	I	V	K	.	.	.	.	S	N	.	.	.	T	.	.	.	T	.	A	.	K	R	.	.	S	I	V	E	.	.	.	.	V

NA

	# of identical sequences	3	9	15	16	30	62	79	82	84	86	100	117	126	189	211	232	234	263	269	285	289	313	328	331	355	364	366	369	374	381	382	385	386	389	394	424
**A/California/04/2009**		**P**	**T**	**M**	**T**	**I**	**V**	**S**	**S**	**K**	**A**	**Y**	**I**	**P**	**N**	**I**	**A**	**V**	**I**	**M**	**S**	**T**	**Q**	**P**	**K**	**N**	**S**	**S**	**N**	**I**	**T**	**G**	**N**	**N**	**I**	**V**	**V**
A/Guatemala/1039/2009		.	.	.	.	.	.	.	.	.	.	.	.	.	.	.	A	.	.	.	.	.	.	.	.	.	.	.	.	.	.	.	.	.	.	.	.
A/sw/GT/CIP049-C1842/2016	5	.	.	.	.	.	.	.	P	.	S	H	M	.	.	.	.	.	.	.	.	.	R	.	R	.	.	.	.	.	.	.	.	.	M	I	.
A/sw/GT/CIP049-C1861/2016	54	.	.	.	.	.	.	.	.	.	.	H	.	.	.	.	.	.	.	.	.	.	.	S	R	.	.	.	.	.	.	.	.	.	M	L	.
A/sw/GT/CIP049-C1972/2016	4	.	.	.	.	.	.	.	.	.	.	H	.	.	.	.	.	.	.	.	.	.	.	S	R	.	.	N	.	.	.	.	.	.	M	L	.
A/sw/GT/CIP049-C1981/2016	1	.	.	.	.	.	.	.	.	.	.	H	.	.	S	.	.	.	.	.	.	.	.	S	R	.	.	.	.	.	.	.	.	.	M	L	.
A/sw/GT/CIP049-C2117/2016	1	.	.	.	.	.	.	.	.	.	.	H	.	.	.	.	.	.	.	.	.	.	.	S	R	.	.	.	T	.	.	.	.	.	M	L	.
A/sw/GT/CIP049-C2299/2016	1	.	.	.	A	.	.	.	.	.	.	H	.	.	.	.	.	.	.	.	.	.	.	S	R	.	.	.	.	.	.	.	.	.	M	L	I
A/sw/GT/CIP049-C2342/2016	1	.	.	.	.	.	L	.	.	.	.	H	.	.	.	.	.	.	.	.	.	.	.	S	R	.	.	.	.	.	.	.	.	.	M	L	.
A/sw/GT/CIP049-C2367/2016	1	.	.	.	.	.	.	.	.	.	.	H	.	.	.	.	.	.	.	.	.	.	.	S	R	.	.	.	.	.	.	.	.	.	.	L	.
A/sw/GT/CIP049-C2380/2016	17	.	.	.	.	.	.	P	.	.	.	H	.	.	.	.	.	.	.	.	.	.	.	S	R	.	.	.	.	.	.	.	.	.	M	L	.
A/sw/GT/CIP049-C2584/2016	15	.	.	.	.	.	.	P	.	.	.	H	.	.	.	.	.	.	V	.	.	.	.	S	R	.	.	.	.	.	.	.	.	.	M	L	.
A/sw/GT/CIP049-C2587/2016	1	.	.	.	.	.	.	P	.	.	.	H	.	H	.	.	.	.	.	.	.	.	.	S	R	.	.	.	.	.	.	.	.	.	M	L	.
A/sw/GT/CIP049-C3615/2018	24	S	A	I	.	T	.	.	.	.	.	H	.	.	.	I	A	I	.	I	.	I	R	.	N	.	N	N	K	.	I	E	K	.	.	.	.
A/sw/GT/CIP049-C3628/2018	5	S	A	I	.	T	.	.	.	.	.	H	.	.	.	I	A	I	.	I	.	I	R	.	N	K	N	N	K	.	I	E	K	.	.	.	.
A/sw/GT/CIP049-C3648/2018	1	S	A	I	.	T	.	.	.	.	.	H	.	.	.	I	A	I	.	I	F	I	R	.	N	.	N	N	K	.	I	E	K	.	.	.	.
A/sw/GT/CIP049-C3805/2018	1	S	A	I	.	T	.	.	.	.	.	H	.	.	.	I	A	I	.	I	.	I	R	.	N	.	N	N	K	.	I	E	K	S	.	.	.
A/sw/GT/CIP049-C3837/2018	1	S	A	I	.	T	.	.	.	.	.	H	.	.	.	I	A	I	.	I	.	I	R	.	N	.	N	N	K	V	I	E	K	.	.	.	.
A/sw/GT/CIP049-C3851/2018	4	S	A	I	.	T	.	.	.	R	.	H	.	.	.	I	A	I	.	I	.	I	R	.	N	.	N	N	K	.	I	E	K	.	.	.	.

PB2

	# of identical sequences	21	64	76	157	165	178	184	194	195	227	300	355	451	453	478	511	524	553	560	584	611	676	683	711	739
**A/California/04/2009**		**T**	**M**	**T**	**K**	**E**	**T**	**A**	**Q**	**D**	**V**	**Q**	**R**	**I**	**S**	**I**	**V**	**T**	**I**	**V**	**V**	**D**	**T**	**T**	**N**	**R**
A/sw/GT/CIP049-C1842/2016	5	.	I	.	.	.	.	T	.	N	.	K	.	.	.	.	.	.	V	.	.	.	.	A	.	.
A/sw/GT/CIP049-C1861/2016	68	.	I	.	.	.	.	T	.	N	.	.	.	.	.	.	.	.	.	.	.	.	.	A	.	.
A/sw/GT/CIP049-C1972/2016	1	.	I	.	.	.	.	T	.	N	.	.	.	.	.	.	.	.	.	.	.	.	.	.	.	.
A/sw/GT/CIP049-C1973/2016	1	.	I	.	.	.	.	T	.	N	.	.	G	.	.	.	.	.	.	.	.	.	.	A	.	.
A/sw/GT/CIP049-C2107/2016	1	.	I	I	.	.	.	T	.	N	.	.	.	.	.	.	.	.	.	I	.	.	.	A	.	.
A/sw/GT/CIP049-C2356/2016	1	.	I	.	.	.	.	T	.	N	.	.	.	.	.	.	.	.	.	.	I	.	.	A	.	.
A/sw/GT/CIP049-C2370/2016	1	.	I	.	.	.	.	T	.	N	.	.	.	.	.	I	.	.	.	.	.	.	.	A	.	.
A/sw/GT/CIP049-C2408/2016	1	.	I	.	.	.	.	T	.	N	.	.	.	.	P	.	.	.	.	.	.	.	.	A	.	.
A/sw/GT/CIP049-C2585/2016	2	.	I	.	.	.	.	T	.	N	.	.	.	T	.	.	.	.	.	.	.	.	.	A	.	.
A/sw/GT/CIP049-C2623/2017	1	.	I	.	.	K	.	T	.	N	.	.	.	.	.	.	.	.	.	.	.	.	.	A	.	.
A/sw/GT/CIP049-C2650/2017	1	.	I	.	.	.	.	T	.	N	.	.	.	.	.	.	.	.	.	.	.	E	.	A	.	.
A/sw/GT/CIP049-C2651/2017	1	N	I	.	.	.	.	T	.	N	.	.	.	.	.	.	.	.	.	.	.	E	.	A	.	.
A/sw/GT/CIP049-C2655/2017	1	N	I	.	.	.	.	T	.	N	.	.	.	.	.	.	.	.	.	.	.	.	.	A	.	.
A/sw/GT/CIP049-C2671/2017	3	.	I	.	.	.	.	T	.	N	I	.	.	.	.	.	.	.	.	.	.	.	.	A	.	.
A/sw/GT/CIP049-C3617/2018	12	.	.	.	.	.	A	.	.	.	.	.	.	.	.	.	I	S	.	.	.	.	I	.	N	.
A/sw/GT/CIP049-C3804/2018	6	.	.	.	N	.	A	.	.	.	.	.	.	.	.	.	I	S	.	.	.	.	I	.	N	.
A/sw/GT/CIP049-C3806/2018	1	.	.	.	N	.	A	.	K	.	.	.	.	.	.	.	I	S	.	.	.	.	I	.	N	.
A/sw/GT/CIP049-C3878/2018	1	.	.	.	N	.	A	.	.	.	.	.	.	.	.	.	I	S	.	.	.	.	I	.	N	Q

PB1

	# of identical sequences	8	40	48	108	114	115	134	152	179	216	317	322	342	353	397	430	455	465	533	652	694	740	743
A/California/04/2009		L	M	Q	L	V	Q	N	S	I	G	M	I	I	K	I	K	N	R	N	A	N	F	I
A/sw/GT/CIP049-C1842/2016	60	.	.	.	.	.	.	.	.	M	S	V	V	V	R	.	.	D	.	.	.	.	.	.
A/sw/GT/CIP049-C1973/2016	1	.	.	.	.	.	K	.	.	M	S	V	A	V	R	.	.	D	.	.	.	.	.	.
A/sw/GT/CIP049-C1982/2016	1	.	.	.	I	.	.	.	.	M	S	V	V	V	R	.	.	D	.	.	.	.	.	.
A/sw/GT/CIP049-C2117/2016	1	.	.	.	.	.	.	.	.	M	S	V	V	V	R	.	.	D	.	.	.	.	.	T
A/sw/GT/CIP049-C2173/2016	2	.	.	.	.	.	.	.	.	M	S	V	V	V	R	.	.	D	.	.	S	.	.	.
A/sw/GT/CIP049-C2367/2016	1	.	.	.	.	.	.	.	.	M	S	V	V	V	R	.	.	D	.	S	.	.	.	.
A/sw/GT/CIP049-C2500/2016	1	P	.	.	.	.	.	.	.	M	S	V	V	V	R	.	.	D	.	.	.	.	.	.
A/sw/GT/CIP049-C2651/2017	1	.	.	.	.	.	.	.	.	M	S	V	V	V	R	.	.	D	.	.	.	.	S	.
A/sw/GT/CIP049-C3627/2018	3	.	M	Q	.	I	.	N	T	M	.	.	.	.	R	.	R	D	K	.	.	H	.	.
A/sw/GT/CIP049-C3804/2018	1	.	M	Q	.	I	.	N	T	M	.	.	.	.	R	M	R	D	K	.	.	H	.	.

PA

	# of identical sequences	20	32	44	55	105	186	224	227	256	261	304	308	327	332	346	364	379	385	387	395	400	405	419	460	470	532	538	556	560	589	650	660	664	688
**A/California/04/2009**		**A**	**T**	**V**	**D**	**F**	**S**	**P**	**E**	**K**	**L**	**L**	**I**	**E**	**P**	**Q**	**S**	**V**	**K**	**V**	**S**	**P**	**S**	**D**	**M**	**L**	**L**	**E**	**Q**	**P**	**L**	**Y**	**A**	**K**	**E**
A/sw/GT/CIP049-C1842/2016	4	T	I	I	N	H	N	S	D	K	.	.	.	A	P	H	.	.	.	.	.	.	.	.	.	.	M	.	.	.	.	F	.	.	G
A/sw/GT/CIP049-C1861/2016	28	T	I	I	N	H	N	S	D	K	.	I	.	A	P	H	.	I	.	.	.	.	.	.	.	.	M	.	.	.	.	F	.	.	.
A/sw/GT/CIP049-C1968/2016	8	T	I	I	N	H	N	S	D	K	.	I	.	A	P	H	.	I	.	.	.	.	.	.	.	.	M	.	.	S	.	F	.	.	.
A/sw/GT/CIP049-C2004/2016	1	T	I	I	N	H	N	S	D	K	.	I	.	A	P	H	.	I	.	.	.	.	.	.	.	.	M	.	.	.	I	F	.	.	.
A/sw/GT/CIP049-C2083/2016	1	T	I	I	N	H	N	S	D	Q	.	I	.	A	P	H	.	I	.	.	.	.	.	.	.	.	M	.	.	.	.	F	.	.	.
A/sw/GT/CIP049-C2173/2016	2	T	I	I	N	H	N	S	D	K	.	I	.	A	P	H	.	I	.	.	.	.	.	.	.	.	M	.	K	.	.	F	.	.	.
A/sw/GT/CIP049-C2213/2016	5	T	I	I	N	H	N	S	D	K	.	I	.	A	P	H	.	I	.	.	.	.	G	.	.	.	M	.	.	.	.	F	.	.	.
A/sw/GT/CIP049-C2224/2016	27	T	I	I	N	H	N	S	D	K	.	I	.	A	P	H	.	I	.	.	.	.	.	.	I	.	M	.	.	.	.	F	.	.	.
A/sw/GT/CIP049-C2256/2016	1	T	I	I	N	H	N	S	D	K	.	I	.	A	P	H	.	I	.	.	.	.	.	.	.	P	M	.	.	.	.	F	.	.	.
A/sw/GT/CIP049-C2258/2016	1	T	I	I	N	H	N	S	D	K	.	I	.	A	P	H	.	I	.	.	.	.	.	.	.	.	M	.	.	.	.	F	G	.	.
A/sw/GT/CIP049-C2408/2016	1	T	I	I	N	H	N	S	D	K	.	I	.	A	P	H	.	I	.	.	.	.	.	.	I	.	M	G	.	.	.	F	.	.	.
A/sw/GT/CIP049-C2547/2016	1	T	I	I	N	H	N	S	D	K	.	I	V	A	P	H	.	I	.	.	.	.	.	.	I	.	M	.	.	.	.	F	.	.	.
A/sw/GT/CIP049-C2622/2017	6	T	L	I	N	H	N	S	D	K	.	I	.	A	P	H	.	I	.	.	.	.	.	.	I	.	M	.	.	.	.	F	.	.	.
A/sw/GT/CIP049-C2636/2017	4	T	I	I	N	H	N	S	D	K	.	I	.	A	P	H	N	I	R	.	.	.	.	.	I	.	M	.	.	.	.	F	.	.	.
A/sw/GT/CIP049-C3615/2018	31	.	.	.	.	L	.	S	.	K	S	.	.	.	.	.	G	.	.	I	G	S	.	N	.	.	.	.	.	.	.	F	.	R	.

NP

	# of identical sequences	31	34	53	186	190	217	242	283	294	353	373	400	401	430	444	455
A/California/04/2009		R	G	D	V	A	V	V	L	E	I	T	K	A	S	V	D
A/sw/GT/CIP049-C1842/2016	11	.	.	E	I	.	.	.	.	.	.	.	R	.	.	.	.
A/sw/GT/CIP049-C1913/2016	55	.	.	E	I	.	.	.	.	.	.	.	R	T	.	.	.
A/sw/GT/CIP049-C2342/2016	1	.	.	E	I	.	I	I	.	.	.	.	R	T	.	.	.
A/sw/GT/CIP049-C2370/2016	1	.	.	E	.	V	.	.	.	.	.	.	R	T	.	.	.
A/sw/GT/CIP049-C2400/2016	1	.	S	E	I	.	.	.	.	.	V	.	R	T	.	.	.
A/sw/GT/CIP049-C2408/2016	31	.	S	E	I	.	.	.	.	.	.	.	R	T	.	.	.
A/sw/GT/CIP049-C2671/2017	1	.	S	E	I	.	.	.	.	X	.	.	R	T	.	.	E
A/sw/GT/CIP049-C2672/2017	3	.	S	E	I	.	.	.	.	.	.	.	R	T	.	.	E
A/sw/GT/CIP049-C3618/2018	35	K	S	E	I	.	.	I	.	.	.	A	.	.	.	I	.
A/sw/GT/CIP049-C3631/2018	1	K	S	E	I	.	.	I	.	.	.	A	.	.	N	I	.
A/sw/GT/CIP049-C3646/2018	1	K	S	E	I	.	.	I	I	.	.	A	.	.	.	I	.

M1

	# of identical sequences	1	13	30	107	149	169	192	201	208	216	225	227	235
**A/California/04/2009**		**M**	**S**	**S**	**I**	**A**	**T**	**M**	**E**	**Q**	**M**	**S**	**A**	**E**
A/sw/GT/CIP049-C1842/2016	12	.	T	.	.	.	.	.	.	.	.	.	.	.
A/sw/GT/CIP049-C1929/2016	3	.	T	.	.	S	.	.	D	.	.	.	.	.
A/sw/GT/CIP049-C2178/2016	1	.	T	.	T	.	.	.	D	.	T	.	.	.
A/sw/GT/CIP049-C2256/2016	1	.	T	N	.	.	.	.	D	.	.	.	.	.
A/sw/GT/CIP049-C2547/2016	98	.	T	.	.	.	.	.	D	.	.	.	.	.
A/sw/GT/CIP049-C2623/2017	1	.	T	.	.	.	.	.	D	.	.	.	.	G
A/sw/GT/CIP049-C3618/2018	41	.	.	.	.	.	I	I	.	R	.	S	A	.

M2

	# of identical sequences	1	18	25	28	33	38	43	48	52	55	58	61	82	95
**A/California/04/2009**		**M**	**R**	**P**	**I**	**I**	**L**	**T**	**F**	**Y**	**F**	**G**	**R**	**S**	**E**
A/sw/GT/CIP049-C1842/2016	39	.	.	.	.	.	.	I	L	.	I	.	K	.	.
A/sw/GT/CIP049-C1861/2016	5	.	.	.	.	V	.	I	L	.	I	.	K	.	.
A/sw/GT/CIP049-C1975/2016	1	.	K	.	.	V	.	I	L	.	I	.	K	.	.
A/sw/GT/CIP049-C2224/2016	1	.	.	.	F	.	.	I	L	.	I	.	K	.	.
A/sw/GT/CIP049-C2299/2016	13	.	.	L	F	.	.	I	L	.	I	.	K	.	.
A/sw/GT/CIP049-C2479/2016	1	.	.	.	F	.	.	I	L	.	I	.	K	.	G
A/sw/GT/CIP049-C2534/2016	1	.	.	.	F	.	M	I	L	.	I	.	K	.	.
A/sw/GT/CIP049-C2547/2016	54	.	.	.	F	.	.	I	L	.	I	.	K	.	.
A/sw/GT/CIP049-C2673/2017	1	.	.	.	F	.	.	I	L	.	I	E	K	.	.
A/sw/GT/CIP049-C3618/2018	41	.	.	.	.	.	.	.	.	H	I	.	K	N	.

NS1

	# of identical sequences	18	52	60	66	72	81	90	91	128	129	155	170	171	181	204	206	213	214	215
A/California/04/2009		**I**	**L**	**V**	**E**	**E**	**I**	**L**	**S**	**I**	**V**	**A**	**T**	**Y**	**L**	**R**	**C**	**S**	**L**	**P**
A/sw/GT/CIP049-C1842/2016	5	.	.	.	D	.	.	.	F	I	.	.	.	.	I	.	.	.	.	.
A/sw/GT/CIP049-C1974/2016	1	.	.	A	.	.	.	.	F	I	.	.	.	.	I	.	.	.	.	.
A/sw/GT/CIP049-C2214/2016	7	.	.	.	.	.	V	.	F	I	.	.	.	.	I	.	.	.	.	.
A/sw/GT/CIP049-C2227/2016	2	V	.	.	.	.	.	.	F	I	.	.	.	.	I	.	.	.	.	.
A/sw/GT/CIP049-C2256/2016	1	.	.	.	.	.	.	.	F	I	.	.	.	.	I	.	.	.	P	.
A/sw/GT/CIP049-C2287/2016	1	.	.	.	.	.	.	F	F	I	.	.	.	.	I	.	.	.	.	.
A/sw/GT/CIP049-C2333/2016	1	.	.	.	.	D	.	.	F	I	.	.	.	.	I	.	.	.	.	.
A/sw/GT/CIP049-C2526/2016	1	.	.	.	.	.	.	.	F	I	.	.	A	.	I	.	.	.	.	.
A/sw/GT/CIP049-C2547/2016	84	.	.	.	.	.	.	.	F	I	.	.	.	.	I	.	.	.	.	.
A/sw/GT/CIP049-C2655/2017	1	.	.	.	.	.	.	.	F	I	I	.	.	.	I	.	.	.	.	.
A/sw/GT/CIP049-C3618/2018	38	.	I	.	.	.	.	.	.	.	.	A	.	H	.	.	S	P	.	S
A/sw/GT/CIP049-C3649/2018	1	.	I	.	.	.	.	.	.	.	.	A	.	H	.	K	S	P	.	S

NEP

	# of identical sequences	12	22	27	34	47	57	74	83	85	86	89	98	99	113
A/California/04/2009		**I**	**G**	**D**	**R**	**E**	**Y**	**E**	**M**	**H**	**R**	**A**	**T**	**F**	**I**
A/sw/GT/CIP049-C1842/2016	5	.	E	.	.	.	.	.	I	.	.	.	.	.	.
A/sw/GT/CIP049-C1861/2016	2	.	.	G	.	.	.	.	I	.	.	.	.	.	.
A/sw/GT/CIP049-C1913/2016	14	.	.	.	.	.	.	.	I	.	.	.	.	.	.
A/sw/GT/CIP049-C1972/2016	4	.	.	G	.	.	.	V	I	.	.	.	.	.	.
A/sw/GT/CIP049-C2026/2016	1	.	.	.	.	.	.	.	I	.	K	.	A	.	.
A/sw/GT/CIP049-C2256/2016	1	.	.	.	.	.	H	.	I	.	K	.	.	.	.
A/sw/GT/CIP049-C2526/2016	1	M	.	.	.	.	.	.	I	.	K	.	.	.	.
A/sw/GT/CIP049-C2547/2016	75	.	.	.	.	.	.	.	I	.	K	.	.	.	.
A/sw/GT/CIP049-C2650/2017	1	.	.	.	.	.	.	.	I	.	K	.	.	L	.
A/sw/GT/CIP049-C3618/2018	38	.	.	.	L	.	.	.	.	H	.	T	.	.	L
A/sw/GT/CIP049-C3649/2018	1	.	.	.	L	K	.	.	.	H	.	T	.	.	L

PA-X

	# of identical sequences	20	32	44	55	105	186	204	205	207	210	212	219	227	229
**A/California/04/2009**		**A**	**T**	**V**	**D**	**F**	**S**	**N**	**L**	**L**	**L**	**A**	**S**	**K**	**L**
A/sw/GT/CIP049-C1842/2016	83	T	I	I	N	H	N	S	.	S	.	.	F	I	S
A/sw/GT/CIP049-C2541/2016	2	T	I	I	N	H	N	S	.	S	.	.	.	I	S
A/sw/GT/CIP049-C2622/2017	6	T	L	I	N	H	N	S	.	S	.	.	F	I	S
A/sw/GT/CIP049-C3615/2018	31	.	.	.	.	L	.	.	S	.	P	E	.	.	.

PA-N155

	# of identical sequences	32	70	73	102	107	150	154	173	178	192	210	225	231	233	241	246	251	265	306	316	378	384	402	406	435	496	506	510	534
**A/California/04/2009**		**S**	**P**	**E**	**K**	**L**	**L**	**I**	**E**	**P**	**Q**	**S**	**V**	**K**	**V**	**S**	**P**	**S**	**D**	**M**	**L**	**L**	**E**	**Q**	**P**	**L**	**Y**	**A**	**K**	**E**
A/sw/GT/CIP049-C1842/2016	4	N	S	D	.	.	.	.	A	.	H	.	.	.	.	.	.	.	.	.	.	M	.	.	.	.	F	.	.	G
A/sw/GT/CIP049-C1861/2016	28	N	S	D	.	.	I	.	A	.	H	.	I	.	.	.	.	.	.	.	.	M	.	.	.	.	F	.	.	.
A/sw/GT/CIP049-C1968/2016	8	N	S	D	.	.	I	.	A	.	H	.	I	.	.	.	.	.	.	.	.	M	.	.	S	.	F	.	.	.
A/sw/GT/CIP049-C2004/2016	1	N	S	D	.	.	I	.	A	.	H	.	I	.	.	.	.	.	.	.	.	M	.	.	.	I	F	.	.	.
A/sw/GT/CIP049-C2083/2016	1	N	S	D	Q	.	I	.	A	.	H	.	I	.	.	.	.	.	.	.	.	M	.	.	.	.	F	.	.	.
A/sw/GT/CIP049-C2173/2016	2	N	S	D	.	.	I	.	A	.	H	.	I	.	.	.	.	.	.	.	.	M	.	K	.	.	F	.	.	.
A/sw/GT/CIP049-C2213/2016	5	N	S	D	.	.	I	.	A	.	H	.	I	.	.	.	.	G	.	.	.	M	.	.	.	.	F	.	.	.
A/sw/GT/CIP049-C2224/2016	34	N	S	D	.	.	I	.	A	.	H	.	I	.	.	.	.	.	.	I	.	M	.	.	.	.	F	.	.	.
A/sw/GT/CIP049-C2256/2016	1	N	S	D	.	.	I	.	A	.	H	.	I	.	.	.	.	.	.	.	P	M	.	.	.	.	F	.	.	.
A/sw/GT/CIP049-C2258/2016	1	N	S	D	.	.	I	.	A	.	H	.	I	.	.	.	.	.	.	.	.	M	.	.	.	.	F	G	.	.
A/sw/GT/CIP049-C2408/2016	1	N	S	D	.	.	I	.	A	.	H	.	I	.	.	.	.	.	.	I	.	M	G	.	.	.	F	.	.	.
A/sw/GT/CIP049-C2547/2016	1	N	S	D	.	.	I	V	A	.	H	.	I	.	.	.	.	.	.	I	.	M	.	.	.	.	F	.	.	.
A/sw/GT/CIP049-C2636/2017	4	N	S	D	.	.	I	.	A	.	H	N	I	R	.	.	.	.	.	I	.	M	.	.	.	.	F	.	.	.
A/sw/GT/CIP049-C3615/2018	31	.	S	.	.	S	.	.	.	S	.	G	.	.	I	G	S	.	N	.	.	.	.	.	.	.	F	.	R	.

PA-N185

	# of identical sequences	5	43	46	75	80	123	127	146	151	165	183	198	204	206	214	219	224	238	279	289	351	357	375	379	408	469	479	483	507
**A/California/04/2009**		**S**	**P**	**E**	**K**	**L**	**L**	**I**	**E**	**P**	**Q**	**S**	**V**	**K**	**V**	**S**	**P**	**S**	**D**	**M**	**L**	**L**	**E**	**Q**	**P**	**L**	**Y**	**A**	**K**	**E**
A/sw/GT/CIP049-C1842/2016	4	N	S	D	.	.	.	.	A	P	H	.	.	.	.	.	.	.	.	.	.	M	.	.	.	.	F	.	.	G
A/sw/GT/CIP049-C1861/2016	28	N	S	D	.	.	I	.	A	P	H	.	I	.	.	.	.	.	.	.	.	M	.	.	.	.	F	.	.	.
A/sw/GT/CIP049-C1968/2016	8	N	S	D	.	.	I	.	A	P	H	.	I	.	.	.	.	.	.	.	.	M	.	.	S	.	F	.	.	.
A/sw/GT/CIP049-C2004/2016	1	N	S	D	.	.	I	.	A	P	H	.	I	.	.	.	.	.	.	.	.	M	.	.	.	I	F	.	.	.
A/sw/GT/CIP049-C2083/2016	1	N	S	D	Q	.	I	.	A	P	H	.	I	.	.	.	.	.	.	.	.	M	.	.	.	.	F	.	.	.
A/sw/GT/CIP049-C2173/2016	2	N	S	D	.	.	I	.	A	P	H	.	I	.	.	.	.	.	.	.	.	M	.	K	.	.	F	.	.	.
A/sw/GT/CIP049-C2213/2016	5	N	S	D	.	.	I	.	A	P	H	.	I	.	.	.	.	G	.	.	.	M	.	.	.	.	F	.	.	.
A/sw/GT/CIP049-C2224/2016	34	N	S	D	.	.	I	.	A	P	H	.	I	.	.	.	.	.	.	I	.	M	.	.	.	.	F	.	.	.
A/sw/GT/CIP049-C2256/2016	1	N	S	D	.	.	I	.	A	P	H	.	I	.	.	.	.	.	.	.	P	M	.	.	.	.	F	.	.	.
A/sw/GT/CIP049-C2258/2016	1	N	S	D	.	.	I	.	A	P	H	.	I	.	.	.	.	.	.	.	.	M	.	.	.	.	F	G	.	.
A/sw/GT/CIP049-C2408/2016	1	N	S	D	.	.	I	.	A	P	H	.	I	.	.	.	.	.	.	I	.	M	G	.	.	.	F	.	.	.
A/sw/GT/CIP049-C2547/2016	1	N	S	D	.	.	I	V	A	P	H	.	I	.	.	.	.	.	.	I	.	M	.	.	.	.	F	.	.	.
A/sw/GT/CIP049-C2636/2017	4	N	S	D	.	.	I	.	A	P	H	N	I	R	.	.	.	.	.	I	.	M	.	.	.	.	F	.	.	.
A/sw/GT/CIP049-C3615/2018	31	.	S	.	.	S	.	.	.	.	.	G	.	.	I	G	S	.	N	.	.	.	.	.	.	.	F	.	R	.

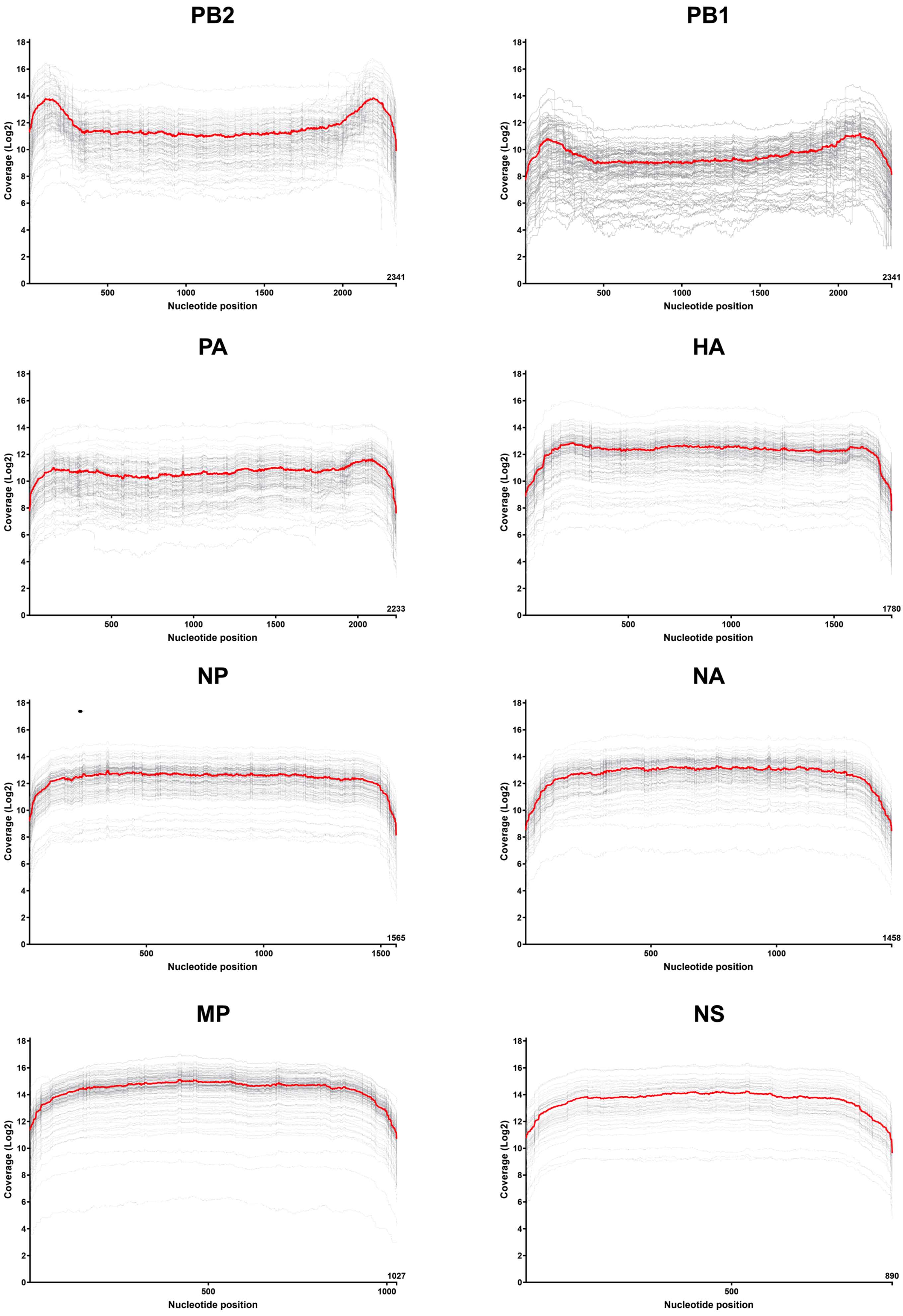

Fig S5. Coverage plot of sequenced samples. Gray lines show the coverage distribution from each individual sample. The red line depicts the geometric mean.

Table S2. Genbank accession numbers for sequences identified from swine samples in Guatemala

Virus	Collection Date (yyyy-mm-dd)	PB2	PB1	PA	HA	NP	NA	M	NS
A/swine/Guatemala/CIP049-C1842/2016	2016-05-12	ON822682	ON822683	ON822684	ON822685	ON822686	ON822687	ON822688	ON822689
A/swine/Guatemala/CIP049-C1861/2016	2016-05-19	ON822690	ON822691	ON822692	ON822693	ON822694	ON822695	ON822696	ON822697
A/swine/Guatemala/CIP049-C1863/2016	2016-05-19	ON822698	ON822699	ON822700	ON822701	ON822702	ON822703	ON822704	ON822705
A/swine/Guatemala/CIP049-C1866/2016	2016-05-19	ON822706	ON822707	ON822708	ON822709	ON822710	ON822711	ON822712	ON822713
A/swine/Guatemala/CIP049-C1869/2016	2016-05-19	---	---	---	---	---	---	ON822118	---
A/swine/Guatemala/CIP049-C1875/2016	2016-05-23	ON822714	ON822715	ON822716	ON822717	ON822718	ON822719	ON822720	ON822721
A/swine/Guatemala/CIP049-C1913/2016	2016-06-06	ON822722	ON822723	ON822724	ON822725	ON822726	ON822727	ON822728	ON822729
A/swine/Guatemala/CIP049-C1914/2016	2016-06-06	ON822730	ON822731	ON822732	ON822733	ON822734	ON822735	ON822736	ON822737
A/swine/Guatemala/CIP049-C1929/2016	2016-06-09	---	---	---	---	---	---	ON822119	---
A/swine/Guatemala/CIP049-C1933/2016	2016-06-09	ON822738	ON822739	ON822740	ON822741	ON822742	ON822743	ON822744	ON822745
A/swine/Guatemala/CIP049-C1934/2016	2016-06-09	ON822746	ON822747	ON822748	ON822749	ON822750	ON822751	ON822752	ON822753
A/swine/Guatemala/CIP049-C1942/2016	2016-06-13	---	ON822120	ON822121	ON822122	ON822123	ON822124	ON822125	ON822126
A/swine/Guatemala/CIP049-C1959/2016	2016-06-16	ON822127	ON822128	---	ON822129	ON822130	ON822131	ON822132	ON822133
A/swine/Guatemala/CIP049-C1968/2016	2016-06-20	ON822754	ON822755	ON822756	ON822757	ON822758	ON822759	ON822760	ON822761
A/swine/Guatemala/CIP049-C1972/2016	2016-06-20	ON822762	ON822763	ON822764	ON822765	ON822766	ON822767	ON822768	ON822769
A/swine/Guatemala/CIP049-C1973/2016	2016-06-20	ON822770	ON822771	ON822772	ON822773	ON822774	ON822775	ON822776	ON822777
A/swine/Guatemala/CIP049-C1974/2016	2016-06-20	---	---	---	ON822134	ON822135	ON822136	ON822137	ON822138
A/swine/Guatemala/CIP049-C1975/2016	2016-06-20	---	ON822139	ON822140	ON822141	ON822142	ON822143	ON822144	ON822145
A/swine/Guatemala/CIP049-C1978/2016	2016-06-23	---	ON822146	---	ON822147	ON822148	ON822149	ON822150	ON822151
A/swine/Guatemala/CIP049-C1980/2016	2016-06-23	---	---	ON822152	ON822153	ON822154	ON822155	ON822156	ON822157
A/swine/Guatemala/CIP049-C1981/2016	2016-06-23	ON822158	---	ON822159	---	ON822160	ON822161	ON822162	ON822163
A/swine/Guatemala/CIP049-C1982/2016	2016-06-23	ON822778	ON822779	ON822780	ON822781	ON822782	ON822783	ON822784	ON822785
A/swine/Guatemala/CIP049-C1983/2016	2016-06-23	ON822786	ON822787	ON822788	ON822789	ON822790	ON822791	ON822792	ON822793
A/swine/Guatemala/CIP049-C1984/2016	2016-06-23	ON822794	ON822795	ON822796	ON822797	ON822798	ON822799	ON822800	ON822801
A/swine/Guatemala/CIP049-C1995/2016	2016-06-28	ON822164	---	---	ON822165	ON822166	ON822167	ON822168	ON822169
A/swine/Guatemala/CIP049-C1996/2016	2016-06-28	ON822802	ON822803	ON822804	ON822805	ON822806	ON822807	ON822808	ON822809
A/swine/Guatemala/CIP049-C1997/2016	2016-06-28	ON822810	ON822811	ON822812	ON822813	ON822814	ON822815	ON822816	ON822817
A/swine/Guatemala/CIP049-C2004/2016	2016-06-30	ON822818	ON822819	ON822820	ON822821	ON822822	ON822823	ON822824	ON822825
A/swine/Guatemala/CIP049-C2008/2016	2016-06-30	ON822826	ON822827	ON822828	ON822829	ON822830	ON822831	ON822832	ON822833
A/swine/Guatemala/CIP049-C2009/2016	2016-06-30	ON822834	ON822835	ON822836	ON822837	ON822838	ON822839	ON822840	ON822841
A/swine/Guatemala/CIP049-C2019/2016	2016-07-05	ON822170	---	ON822171	ON822172	ON822173	ON822174	ON822175	ON822176
A/swine/Guatemala/CIP049-C2023/2016	2016-07-05	---	---	---	ON822177	---	---	ON822178	ON822179
A/swine/Guatemala/CIP049-C2026/2016	2016-07-05	---	ON822180	ON822181	ON822182	ON822183	ON822184	ON822185	ON822186
A/swine/Guatemala/CIP049-C2031/2016	2016-07-07	ON822842	ON822843	ON822844	ON822845	ON822846	ON822847	ON822848	ON822849
A/swine/Guatemala/CIP049-C2032/2016	2016-07-07	ON822850	ON822851	ON822852	ON822853	ON822854	ON822855	ON822856	ON822857
A/swine/Guatemala/CIP049-C2071/2016	2016-07-20	ON822858	ON822859	ON822860	ON822861	ON822862	ON822863	ON822864	ON822865
A/swine/Guatemala/CIP049-C2078/2016	2016-07-21	---	ON822187	---	ON822188	ON822189	ON822190	ON822191	ON822192
A/swine/Guatemala/CIP049-C2083/2016	2016-07-21	ON822866	ON822867	ON822868	ON822869	ON822870	ON822871	ON822872	ON822873
A/swine/Guatemala/CIP049-C2101/2016	2016-07-28	ON822193	ON822194	---	ON822195	ON822196	ON822197	ON822198	ON822199
A/swine/Guatemala/CIP049-C2107/2016	2016-07-28	ON822874	ON822875	ON822876	ON822877	ON822878	ON822879	ON822880	ON822881
A/swine/Guatemala/CIP049-C2114/2016	2016-08-02	---	ON822200	ON822201	ON822202	ON822203	ON822204	ON822205	ON822206

A/swine/Guatemala/CIP049-C2117/2016	2016-08-02	ON822882	ON822883	ON822884	ON822885	ON822886	ON822887	ON822888	ON822889
A/swine/Guatemala/CIP049-C2168/2016	2016-08-18	ON822890	ON822891	ON822892	ON822893	ON822894	ON822895	ON822896	ON822897
A/swine/Guatemala/CIP049-C2173/2016	2016-08-18	ON822898	ON822899	ON822900	ON822901	ON822902	ON822903	ON822904	ON822905
A/swine/Guatemala/CIP049-C2176/2016	2016-08-18	ON822207	---	ON822208	ON822209	ON822210	ON822211	ON822212	ON822213
A/swine/Guatemala/CIP049-C2178/2016	2016-08-18	---	---	---	ON822214	ON822215	ON822216	ON822217	ON822218
A/swine/Guatemala/CIP049-C2213/2016	2016-08-29	ON822906	ON822907	ON822908	ON822909	ON822910	ON822911	ON822912	ON822913
A/swine/Guatemala/CIP049-C2214/2016	2016-08-29	---	---	ON822219	ON822220	---	ON822221	ON822222	ON822223
A/swine/Guatemala/CIP049-C2216/2016	2016-08-29	---	---	---	ON822224	ON822225	ON822226	ON822227	ON822228
A/swine/Guatemala/CIP049-C2222/2016	2016-08-31	---	ON822229	ON822230	ON822231	ON822232	ON822233	ON822234	ON822235
A/swine/Guatemala/CIP049-C2223/2016	2016-08-31	ON822236	ON822237	---	ON822238	ON822239	ON822240	ON822241	ON822242
A/swine/Guatemala/CIP049-C2224/2016	2016-08-31	---	---	ON822243	ON822244	ON822245	ON822246	ON822247	ON822248
A/swine/Guatemala/CIP049-C2225/2016	2016-08-31	ON822914	ON822915	ON822916	ON822917	ON822918	ON822919	ON822920	ON822921
A/swine/Guatemala/CIP049-C2226/2016	2016-08-31	---	---	---	---	---	---	ON822249	ON822250
A/swine/Guatemala/CIP049-C2227/2016	2016-08-31	ON822922	ON822923	ON822924	ON822925	ON822926	ON822927	ON822928	ON822929
A/swine/Guatemala/CIP049-C2228/2016	2016-08-31	ON822930	ON822931	ON822932	ON822933	ON822934	ON822935	ON822936	ON822937
A/swine/Guatemala/CIP049-C2230/2016	2016-08-31	ON822938	ON822939	ON822940	ON822941	ON822942	ON822943	ON822944	ON822945
A/swine/Guatemala/CIP049-C2231/2016	2016-08-31	ON822251	---	---	ON822252	ON822253	ON822254	ON822255	ON822256
A/swine/Guatemala/CIP049-C2232/2016	2016-08-31	ON822946	ON822947	ON822948	ON822949	ON822950	ON822951	ON822952	ON822953
A/swine/Guatemala/CIP049-C2233/2016	2016-08-31	ON822954	ON822955	ON822956	ON822957	ON822958	ON822959	ON822960	ON822961
A/swine/Guatemala/CIP049-C2256/2016	2016-09-07	ON822962	ON822963	ON822964	ON822965	ON822966	ON822967	ON822968	ON822969
A/swine/Guatemala/CIP049-C2258/2016	2016-09-12	ON822257	---	ON822258	ON822259	ON822260	ON822261	ON822262	ON822263
A/swine/Guatemala/CIP049-C2287/2016	2016-09-19	ON822970	ON822971	ON822972	ON822973	ON822974	ON822975	ON822976	ON822977
A/swine/Guatemala/CIP049-C2289/2016	2016-09-19	---	---	---	ON822264	ON822265	---	ON822266	ON822267
A/swine/Guatemala/CIP049-C2299/2016	2016-09-21	ON822268	---	ON822269	ON822270	ON822271	ON822272	ON822273	ON822274
A/swine/Guatemala/CIP049-C2300/2016	2016-09-21	---	ON822275	---	---	---	---	ON822276	---
A/swine/Guatemala/CIP049-C2322/2016	2016-09-28	ON822978	ON822979	ON822980	ON822981	ON822982	ON822983	ON822984	ON822985
A/swine/Guatemala/CIP049-C2331/2016	2016-10-03	ON822986	ON822987	ON822988	ON822989	ON822990	ON822991	ON822992	ON822993
A/swine/Guatemala/CIP049-C2333/2016	2016-10-03	ON822277	---	ON822278	ON822279	ON822280	ON822281	ON822282	ON822283
A/swine/Guatemala/CIP049-C2334/2016	2016-10-03	ON822284	---	ON822285	ON822286	ON822287	ON822288	ON822289	ON822290
A/swine/Guatemala/CIP049-C2335/2016	2016-10-03	---	---	---	---	---	---	ON822291	---
A/swine/Guatemala/CIP049-C2342/2016	2016-10-05	ON822994	ON822995	ON822996	ON822997	ON822998	ON822999	ON823000	ON823001
A/swine/Guatemala/CIP049-C2356/2016	2016-10-10	ON822292	---	---	ON822293	ON822294	ON822295	ON822296	---
A/swine/Guatemala/CIP049-C2367/2016	2016-10-12	---	ON822297	ON822298	ON822299	ON822300	ON822301	ON822302	ON822303
A/swine/Guatemala/CIP049-C2370/2016	2016-10-12	ON823002	ON823003	ON823004	ON823005	ON823006	ON823007	ON823008	ON823009
A/swine/Guatemala/CIP049-C2380/2016	2016-10-17	ON823010	ON823011	ON823012	ON823013	ON823014	ON823015	ON823016	ON823017
A/swine/Guatemala/CIP049-C2400/2016	2016-10-19	ON822304	---	ON822305	ON822306	ON822307	ON822308	ON822309	ON822310
A/swine/Guatemala/CIP049-C2408/2016	2016-10-24	ON822311	---	ON822312	ON822313	ON822314	---	ON822315	ON822316
A/swine/Guatemala/CIP049-C2415/2016	2016-10-26	---	ON822317	ON822318	ON822319	ON822320	ON822321	ON822322	ON822323
A/swine/Guatemala/CIP049-C2420/2016	2016-10-26	---	---	ON822324	---	---	---	ON822325	---
A/swine/Guatemala/CIP049-C2477/2016	2016-11-14	ON823018	ON823019	ON823020	ON823021	ON823022	ON823023	ON823024	ON823025
A/swine/Guatemala/CIP049-C2478/2016	2016-11-14	ON822326	---	ON822327	ON822328	ON822329	ON822330	ON822331	ON822332
A/swine/Guatemala/CIP049-C2479/2016	2016-11-14	ON822333	---	ON822334	ON822335	ON822336	ON822337	ON822338	ON822339
A/swine/Guatemala/CIP049-C2480/2016	2016-11-14	ON822340	---	ON822341	ON822342	ON822343	ON822344	ON822345	---
A/swine/Guatemala/CIP049-C2481/2016	2016-11-14	---	---	---	ON822346	ON822347	---	ON822348	---
A/swine/Guatemala/CIP049-C2500/2016	2016-11-21	ON823026	ON823027	ON823028	ON823029	ON823030	ON823031	ON823032	ON823033
A/swine/Guatemala/CIP049-C2501/2016	2016-11-21	ON823034	ON823035	ON823036	ON823037	ON823038	ON823039	ON823040	ON823041

A/swine/Guatemala/CIP049-C2523/2016	2016-11-30	ON822349	---	ON822350	ON822351	ON822352	ON822353	ON822354	---
A/swine/Guatemala/CIP049-C2526/2016	2016-11-30	ON822355	---	ON822356	ON822357	ON822358	---	ON822359	ON822360
A/swine/Guatemala/CIP049-C2534/2016	2016-12-01	---	---	---	ON822361	ON822362	ON822363	ON822364	ON822365
A/swine/Guatemala/CIP049-C2536/2016	2016-12-01	---	---	---	---	---	---	ON822366	---
A/swine/Guatemala/CIP049-C2541/2016	2016-12-01	ON822367	---	ON822368	ON822369	ON822370	ON822371	ON822372	ON822373
A/swine/Guatemala/CIP049-C2546/2016	2016-12-05	ON822374	---	ON822375	ON822376	ON822377	ON822378	ON822379	ON822380
A/swine/Guatemala/CIP049-C2547/2016	2016-12-05	ON822381	---	ON822382	ON822383	---	---	ON822384	ON822385
A/swine/Guatemala/CIP049-C2553/2016	2016-12-05	ON822386	---	ON822387	ON822388	ON822389	ON822390	ON822391	ON822392
A/swine/Guatemala/CIP049-C2563/2016	2016-12-07	ON823042	ON823043	ON823044	ON823045	ON823046	ON823047	ON823048	ON823049
A/swine/Guatemala/CIP049-C2584/2016	2016-12-14	ON822393	---	ON822394	ON822395	ON822396	ON822397	ON822398	ON822399
A/swine/Guatemala/CIP049-C2585/2016	2016-12-14	ON822400	---	ON822401	ON822402	ON822403	ON822404	ON822405	ON822406
A/swine/Guatemala/CIP049-C2587/2016	2016-12-14	ON823050	ON823051	ON823052	ON823053	ON823054	ON823055	ON823056	ON823057
A/swine/Guatemala/CIP049-C2588/2016	2016-12-14	---	---	ON822407	ON822408	ON822409	ON822410	ON822411	ON822412
A/swine/Guatemala/CIP049-C2601/2016	2016-12-19	ON823058	ON823059	ON823060	ON823061	ON823062	ON823063	ON823064	ON823065
A/swine/Guatemala/CIP049-C2609/2016	2016-12-22	---	ON822413	ON822414	ON822415	ON822416	ON822417	ON822418	ON822419
A/swine/Guatemala/CIP049-C2610/2016	2016-12-22	ON822420	---	---	---	---	---	ON822421	---
A/swine/Guatemala/CIP049-C2622/2017	2017-01-10	ON822422	---	ON822423	ON822424	ON822425	ON822426	ON822427	ON822428
A/swine/Guatemala/CIP049-C2623/2017	2017-01-10	ON823066	ON823067	ON823068	ON823069	ON823070	ON823071	ON823072	ON823073
A/swine/Guatemala/CIP049-C2632/2017	2017-01-12	ON822429	---	---	ON822430	---	ON822431	ON822432	ON822433
A/swine/Guatemala/CIP049-C2633/2017	2017-01-12	---	---	---	---	ON822434	---	ON822435	ON822436
A/swine/Guatemala/CIP049-C2636/2017	2017-01-12	ON822437	---	ON822438	ON822439	ON822440	ON822441	ON822442	ON822443
A/swine/Guatemala/CIP049-C2650/2017	2017-01-18	ON822444	---	ON822445	ON822446	ON822447	ON822448	ON822449	ON822450
A/swine/Guatemala/CIP049-C2651/2017	2017-01-18	ON823074	ON823075	ON823076	ON823077	ON823078	ON823079	ON823080	ON823081
A/swine/Guatemala/CIP049-C2655/2017	2017-01-18	ON822451	---	ON822452	ON822453	ON822454	ON822455	ON822456	ON822457
A/swine/Guatemala/CIP049-C2670/2017	2017-01-23	ON823082	ON823083	ON823084	ON823085	ON823086	ON823087	ON823088	ON823089
A/swine/Guatemala/CIP049-C2671/2017	2017-01-23	ON822458	---	ON822459	ON822460	---	ON822461	ON822462	---
A/swine/Guatemala/CIP049-C2672/2017	2017-01-23	ON823090	ON823091	ON823092	ON823093	ON823094	ON823095	ON823096	ON823097
A/swine/Guatemala/CIP049-C2673/2017	2017-01-23	ON823098	ON823099	ON823100	ON823101	ON823102	ON823103	ON823104	ON823105
A/swine/Guatemala/CIP049-C2680/2017	2017-01-25	---	---	---	ON822463	ON822464	ON822465	ON822466	ON822467
A/swine/Guatemala/CIP049-C3615/2018	2018-01-29	---	---	ON822468	ON822469	ON822470	ON822471	ON822472	ON822473
A/swine/Guatemala/CIP049-C3616/2018	2018-01-29	---	---	---	---	---	---	ON822474	ON822475
A/swine/Guatemala/CIP049-C3617/2018	2018-01-29	ON822476	---	ON822477	ON822478	ON822479	ON822480	ON822481	ON822482
A/swine/Guatemala/CIP049-C3618/2018	2018-01-29	---	---	---	ON822483	ON822484	ON822485	ON822486	ON822487
A/swine/Guatemala/CIP049-C3619/2018	2018-01-29	---	---	ON822488	ON822489	ON822490	ON822491	ON822492	ON822493
A/swine/Guatemala/CIP049-C3620/2018	2018-01-31	---	---	ON822494	ON822495	ON822496	ON822497	ON822498	ON822499
A/swine/Guatemala/CIP049-C3627/2018	2018-01-31	ON823106	ON823107	ON823108	ON823109	ON823110	ON823111	ON823112	ON823113
A/swine/Guatemala/CIP049-C3628/2018	2018-01-31	---	---	---	ON822500	ON822501	ON822502	ON822503	ON822504
A/swine/Guatemala/CIP049-C3629/2018	2018-01-31	ON823114	ON823115	ON823116	ON823117	ON823118	ON823119	ON823120	ON823121
A/swine/Guatemala/CIP049-C3630/2018	2018-01-31	ON822505	---	ON822506	ON822507	ON822508	ON822509	ON822510	ON822511
A/swine/Guatemala/CIP049-C3631/2018	2018-01-31	ON822512	---	ON822513	ON822514	ON822515	---	ON822516	ON822517
A/swine/Guatemala/CIP049-C3632/2018	2018-02-05	---	---	ON822518	ON822519	ON822520	ON822521	ON822522	ON822523
A/swine/Guatemala/CIP049-C3633/2018	2018-02-05	ON823122	ON823123	ON823124	ON823125	ON823126	ON823127	ON823128	ON823129
A/swine/Guatemala/CIP049-C3635/2018	2018-02-05	---	---	ON822524	ON822525	ON822526	ON822527	ON822528	ON822529
A/swine/Guatemala/CIP049-C3644/2018	2018-02-07	ON822530	---	ON822531	ON822532	ON822533	ON822534	ON822535	ON822536
A/swine/Guatemala/CIP049-C3645/2018	2018-02-07	ON822537	---	ON822538	ON822539	ON822540	ON822541	ON822542	ON822543
A/swine/Guatemala/CIP049-C3646/2018	2018-02-07	ON822544	---	ON822545	ON822546	ON822547	ON822548	ON822549	ON822550

A/swine/Guatemala/CIP049-C3648/2018	2018-02-07	ON822551	---	ON822552	ON822553	ON822554	ON822555	ON822556	ON822557
A/swine/Guatemala/CIP049-C3649/2018	2018-02-07	---	---	ON822558	ON822559	ON822560	ON822561	ON822562	ON822563
A/swine/Guatemala/CIP049-C3658/2018	2018-02-12	---	---	---	---	---	ON822564	ON822565	---
A/swine/Guatemala/CIP049-C3661/2018	2018-02-12	---	---	---	ON822566	ON822567	ON822568	ON822569	ON822570
A/swine/Guatemala/CIP049-C3789/2018	2018-04-02	---	---	ON822571	ON822572	ON822573	ON822574	ON822575	ON822576
A/swine/Guatemala/CIP049-C3791/2018	2018-04-02	---	---	---	ON822577	ON822578	ON822579	ON822580	ON822581
A/swine/Guatemala/CIP049-C3792/2018	2018-04-02	---	---	ON822582	ON822583	ON822584	---	ON822585	ON822586
A/swine/Guatemala/CIP049-C3800/2018	2018-04-04	---	---	---	ON822587	---	ON822588	ON822589	---
A/swine/Guatemala/CIP049-C3802/2018	2018-04-04	---	---	ON822590	---	ON822591	ON822592	ON822593	ON822594
A/swine/Guatemala/CIP049-C3803/2018	2018-04-04	---	---	ON822595	ON822596	ON822597	ON822598	ON822599	ON822600
A/swine/Guatemala/CIP049-C3804/2018	2018-04-04	ON823130	ON823131	ON823132	ON823133	ON823134	ON823135	ON823136	ON823137
A/swine/Guatemala/CIP049-C3805/2018	2018-04-04	ON822601	---	---	ON822602	ON822603	ON822604	ON822605	ON822606
A/swine/Guatemala/CIP049-C3806/2018	2018-04-04	ON822607	---	ON822608	ON822609	ON822610	ON822611	ON822612	ON822613
A/swine/Guatemala/CIP049-C3836/2018	2018-04-16	ON822614	---	ON822615	ON822616	ON822617	ON822618	ON822619	ON822620
A/swine/Guatemala/CIP049-C3837/2018	2018-04-16	ON822621	---	ON822622	ON822623	ON822624	ON822625	ON822626	ON822627
A/swine/Guatemala/CIP049-C3839/2018	2018-04-16	ON822628	---	ON822629	ON822630	---	---	ON822631	ON822632
A/swine/Guatemala/CIP049-C3840/2018	2018-04-16	ON822633	---	ON822634	ON822635	ON822636	ON822637	ON822638	ON822639
A/swine/Guatemala/CIP049-C3841/2018	2018-04-16	---	---	---	ON822640	ON822641	---	ON822642	ON822643
A/swine/Guatemala/CIP049-C3851/2018	2018-04-18	---	---	---	ON822644	ON822645	ON822646	ON822647	ON822648
A/swine/Guatemala/CIP049-C3852/2018	2018-04-18	ON822649	---	ON822650	ON822651	ON822652	ON822653	ON822654	ON822655
A/swine/Guatemala/CIP049-C3853/2018	2018-04-18	ON822656	---	ON822657	ON822658	ON822659	ON822660	ON822661	ON822662
A/swine/Guatemala/CIP049-C3854/2018	2018-04-18	---	---	ON822663	ON822664	ON822665	ON822666	ON822667	ON822668
A/swine/Guatemala/CIP049-C3866/2018	2018-04-23	---	---	ON822669	ON822670	ON822671	ON822672	ON822673	ON822674
A/swine/Guatemala/CIP049-C3878/2018	2018-04-25	ON822675	---	ON822676	ON822677	ON822678	ON822679	ON822680	ON822681
